# Organelle tethering, pore formation and SNARE compensation in the late endocytic pathway

**DOI:** 10.1242/jcs.255463

**Published:** 2021-05-27

**Authors:** Luther J. Davis, Nicholas A. Bright, James R. Edgar, Michael D. J. Parkinson, Lena Wartosch, Judith Mantell, Andrew A. Peden, J. Paul Luzio

**Affiliations:** 1 Cambridge Institute for Medical Research (CIMR) and Department of Clinical Biochemistry, University of Cambridge School of Clinical Medicine, The Keith Peters Building, Cambridge Biomedical Campus, Hills Road, Cambridge CB2 0XY, UK; 2 School of Biochemistry, University of Bristol, Medical Sciences Building, University Walk, Bristol BS81TD, UK; 3 Wolfson Bioimaging Facility, University of Bristol, Medical Sciences Building, University Walk, Bristol BS81TD, UK; 4 Department of Biomedical Science & Centre for Membrane Interactions and Dynamics (CMIAD), The University of Sheffield, Western Bank, Sheffield S10 2TN, UK

**Keywords:** Lysosome, Endosome, Membrane fusion

## Abstract

To provide insights into the kiss-and-run and full fusion events resulting in endocytic delivery to lysosomes, we investigated conditions causing increased tethering and pore formation between late endocytic organelles in HeLa cells. Knockout of the soluble N-ethylmaleimide-sensitive factor attachment protein receptors (SNAREs) VAMP7 and VAMP8 showed, by electron microscopy, the accumulation of tethered lysosome-associated membrane protein (LAMP)-carrier vesicles around multivesicular bodies, as well as the appearance of ‘hourglass’ profiles of late endocytic organelles attached by filamentous tethers, but did not prevent endocytic delivery to lysosomal hydrolases. Subsequent depletion of the SNARE YKT6 reduced this delivery, consistent with it compensating for the absence of VAMP7 and VAMP8. We also investigated filamentous tethering between multivesicular bodies and enlarged endolysosomes following depletion of charged multi-vesicular body protein 6 (CHMP6), and provide the first evidence that pore formation commences at the edge of tether arrays, with pore expansion required for full membrane fusion.

## Abstract (precis)

**Summary:** Endocytic delivery to lysosomes by kiss and run/membrane fusion entails pore formation commencing at the edge of tether arrays, and demonstrates SNARE redundancy and compensation.

## INTRODUCTION

The delivery of endocytosed macromolecules to lysosomal hydrolases in mammalian cells requires kiss-and-run and/or complete fusion events between late endosomes, also known as multi-vesicular bodies (MVBs), and lysosomes to form acidic endolysosomes, in which hydrolytic digestion occurs (Luzio et al., 2007; Huotari and Helenius, 2011; Bright et al., 2016). Like other membrane fusion events on the secretory and endocytic pathways, endosome-lysosome fusion requires three sequential steps: (1) tethering, (2) formation of the trans soluble N-ethylmaleimide-sensitive factor-attachment protein receptor (SNARE) complex and (3) fusion of the phospholipid bilayer (Luzio et al., 2007). Tethering includes a requirement for the homotypic fusion and vacuole protein sorting (HOPS) multi-subunit protein complex, likely to be analogous to its role in vacuole fusion in yeast (Hickey and Wickner, 2010; Balderhaar and Ungermann, 2013; Zick and Wickner, 2014). Antibody inhibition studies in cell-free systems have identified the three glutamine type (Q)-SNAREs, i.e. syntaxin 7, vesicle transport through interaction with t-SNAREs 1B (VTI1B) and syntaxin 8, plus two different arginine type (R)-SNAREs, i.e. vesicle-associated membrane proteins 7 and 8 (VAMP7 and VAMP8), as being required for membrane fusion events in the late endocytic pathway (Antonin et al., 2000; Ward et al., 2000; Pryor et al., 2004). VAMP7 has also been implicated in a variety of other intracellular fusion events (reviewed by Daste et al., 2015), including lysosome fusion with the plasma membrane and autophagosomes, and the fusion of trans-Golgi network-derived lysosome-associated membrane protein (LAMP) carriers with late endosomes, a process required for lysosome biogenesis (Pols et al., 2013a). However, the lack of any obvious endocytic/lysosomal phenotype in VAMP7- or VAMP8-knockout mice (Wang et al., 2004; Sato et al., 2011; Danglot et al., 2012), together with little or no effect on endocytic delivery to lysosomes following knockdown of VAMP7, VAMP8 or both in cultured cells (Pols et al., 2013b), suggests that our current understanding of the role played by these R-SNAREs in the late endocytic pathway is far from complete.

In addition to our incomplete knowledge of SNARE function in the late endocytic pathway, there is neither a clear description of how pores physically form to allow content mixing between tethered late endocytic organelles in mammalian cells, nor of the relationship of pore formation to tethered regions. This contrasts with the description of the vertex ring fusion of yeast vacuoles (Wang et al., 2002) and is despite a number of electron microscopy (EM) observations of physical tethers between late endocytic organelles in mammalian cells, after a variety of manipulations (Futter et al., 1996; Bright et al., 1997; Row et al., 2006). These physical tethers can have the appearance of ordered arrays of filaments, most notably those sometimes seen between endosomes and lysosomes in cells that had endocytosed horseradish peroxidase (HRP)-conjugated epidermal growth factor (EGF), and in which endosome–lysosome fusion was subsequently inhibited by incubation with diaminobenzidine and H_2_O_2_ (Futter et al., 1996).

To gain further insight into the relationship between tethers and pore formation during the fusion of late endocytic organelles, we have explored two conditions in which increased tethering might be expected in HeLa cells. These conditions were the double knockout of VAMP7 and VAMP8, as well as the knockdown/depletion of the endosomal sorting complex required for transport-III (ESCRT-III) protein charged multi-vesicular body (MVB) protein 6 (CHMP6). Double knockout of VAMP7 and VAMP8 (VAMP7+VAMP8) should affect the morphology, tethering and function of late endocytic organelles because tethering precedes the formation of trans-SNARE complexes. Similarly, increased organelle tethering was also expected following CHMP6 knockdown, since this treatment results in clusters of MVBs closely associated with enlarged, ubiquitylated, LAMP1-positive endolysosomal compartments (Parkinson et al., 2015). This phenotype is likely to be caused by the inefficient sorting of some ubiquitylated cargoes into intraluminal vesicles (ILVs) and their resultant accumulation on the limiting membrane of MVBs, interfering with efficient fusion between endosomes and lysosomes, as well as disruption of subsequent fission events (Parkinson et al., 2015). Our present observations provide the first insights of where pores form relative to regions of filamentous tethering, as well as evidence for R-SNARE compensation in the late endosome–lysosome fusion pathway of mammalian cells.

## RESULTS

### Knocking out VAMP7 and VAMP8 alters the morphology of late endocytic compartments but not endocytic delivery to lysosomes

**Fig. 1.**
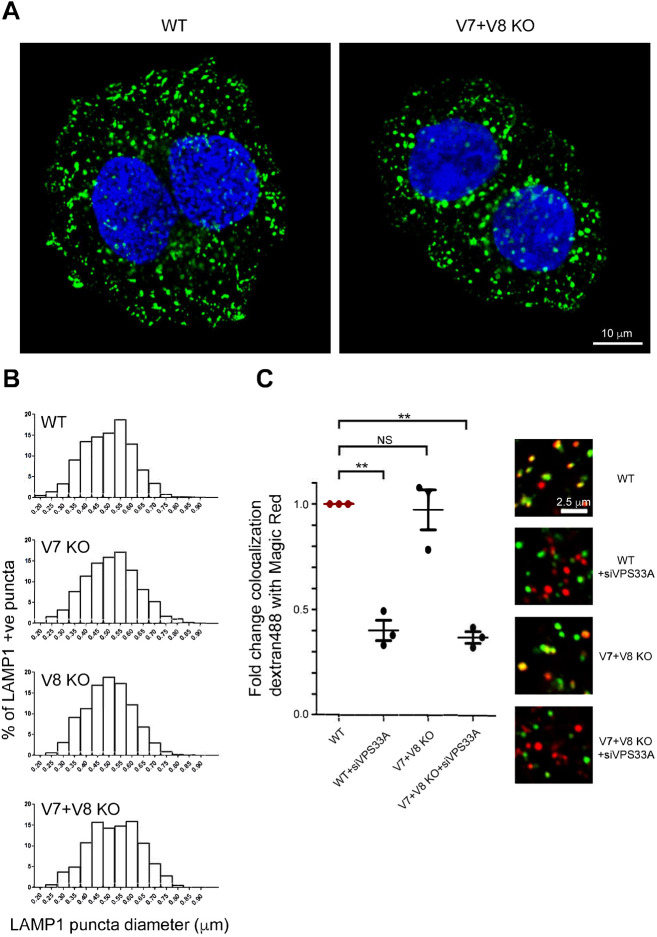
**Effect of knocking out VAMP7 and/or VAMP8 on the morphology of lysosomes and endocytic delivery to lysosomes.** (A) Confocal immunofluorescence microscopy images of wild-type (WT) and VAMP7+VAMP8 double knockout (V7+V8 KO) HeLa cells showing LAMP1-positive organelles (green). Nuclei were labelled with Hoechst 33342 (Blue). (B) Size distribution of LAMP1-positive organelles in single confocal sections (0.7 µm thick) of ten LAMP1-stained cells analysed with Imaris software, following single or double knockout of VAMP7 and VAMP8. Histograms show the percentage of LAMP1-positive puncta in each diameter bin, increasing in increments of 0.05 µm. Mean diameters ±s.e.m. of LAMP1-positive puncta were WT, 497±3 nm (1602 puncta); V7 KO, 512±3 nm (1812); V8 KO, 499±2 nm (1746); V7+V8 KO, 524±4 nm (896). The size for V7 KO and V7+V8 KO is significantly different from that of wild type (P<0.0001). (C) Colocalization (Manders’ coefficient) of endocytosed fluorescent dextran (loaded for 2 h, chased for 1 h) with hydrolysed cathepsin B Magic Red substrate using images captured by live-cell confocal microscopy in VAMP7+VAMP8 double knockout HeLa cells. Means±s.e.m. of three independent experiments with five fields each, ≥30 cells total per condition. **P<0.01 (two-tailed unpaired *t*-test); NS, not significant. Knockdown of VPS33A (+siVPS33a) was used as a positive control to block endocytic delivery to the Magic Red-positive organelles. Representative confocal microscopy images are shown on the right.

Using CRISPR/Cas9 technology, we knocked out either VAMP7 or VAMP8, or both in cultured HeLa cells. We then selected and expanded clonal cell lines in which the relevant genes were disrupted, which was confirmed by DNA sequencing, and found no detectable protein by immunoblotting (Fig. S1A). In selected knockout cell lines, we examined the distribution of a variety of subcellular markers by using confocal immunofluorescence microscopy. In the VAMP7+VAMP8 double knockout cells, we observed a subtle alteration in the distribution of the late endosomal/lysosomal marker LAMP1, with what appeared to be a higher proportion of larger than normal puncta (Fig. 1A). The altered distribution of LAMP1 labelling in these cells was confirmed by quantifying the number of LAMP1-positive puncta of different sizes per cell profile (Fig. 1B). A VAMP7 knockout cell line also showed a change in size distribution of LAMP1-positive puncta, but this was less pronounced (Fig. 1B). In contrast to the change in LAMP1-positive structures, we saw no obvious alteration in the morphology of early endosome antigen 1 (EEA1)-positive early endosomal compartments (Fig. S1B). We detected no change in the steady-state localization of the endosome/trans-Golgi network (TGN) recycling receptor cation independent mannose 6-phosphate receptor (ciMPR) or the morphology of the TGN observed by staining TGN46 in any of the knockout cell lines, when compared with control HeLa cells (Fig. S1B). Moreover, we saw no obvious alteration of autophagy assessed by immunoblotting of lipidated microtubule-associated protein 1 light chain 3B (MAP1LC3B or LC3B, lipdated form denoted LC3-II; Fig. S1C). The alteration in the distribution of labelled LAMP1 in the double knockout cells was partly reversed by overexpression of VAMP7, which resulted in a shift towards smaller LAMP1-positive puncta (Fig. S1D,E).

We assessed endocytic delivery to lysosomes in the knockout cell lines by using a previously published protocol (Pols et al., 2013b; Wartosch et al., 2015). This allowed us to visualize and quantify the delivery of endocytosed fluorescently labelled dextran (2 h pulse/1 h chase protocol) to enzymatically active endolysosomal/lysosomal compartments, identified as containing the red fluorescent Cresyl Violet hydrolysis product of Magic Red, a substrate for the lysosomal acid hydrolase cathepsin B. Paradoxically, in view of previous cell-free, antibody-inhibition experiments showing the role of VAMP7 and VAMP8 in late endocytic pathway fusion events (Antonin et al., 2000; Ward et al., 2000; Pryor et al., 2004), we saw no effect when knocking out VAMP7 and VAMP8 together (Fig. 1C), or either R-SNARE alone (Fig. S1F), implying the presence of a compensatory R-SNARE. In these experiments, knockdown of VPS33A was used as a positive control (Fig. 1C) because we have previously shown that depletion of this or other proteins of the HOPS complex blocks endocytic delivery to the Magic Red-positive organelles (Wartosch et al., 2015). When the late endocytic compartments in knockout cell lines were loaded to steady-state by using fluorescent dextran in a 4 h pulse/20 h chase protocol (Bright et al., 2016), there was no change in the proportion of these compartments that were acidic or proteolytically active – as defined by LysoTracker Green or Magic Red staining – when compared with control HeLa cells (Fig. S1G,H).

The discovery that double VAMP7+VAMP8 knockout altered the distribution of LAMP1 labelling, but did not affect endocytic delivery to lysosomes, led us to investigate further the morphology of the late endocytic compartments at higher resolution using EM (below) and to establish the identity of a compensatory R-SNARE functioning when neither VAMP7 nor VAMP8 are present (see further below).

### Knockout of VAMP7+VAMP8 increases tethering of vesicles and organelles in the late endocytic pathway

Our EM studies required the identification of late endocytic organelles including late endosomes, endolysosomes and lysosomes in the absence of immunolabelling. The formation of late endosomes from early endosomes is a maturation process accompanied by an exchange of Rab5 for Rab7 and the accumulation of ILVs (Huotari and Helenius, 2011). Various studies (reviewed by Klumperman and Raposo, 2014), have suggested that the switch from early to late endosomes occurs when as few as five to eight ILVs accumulate, with an increasing number forming as the late endosome matures. Thus, an EM section that contains even relatively few ILVs is likely to be a section through a late endosome; albeit one that has not completed its maturation. Other physical features, such as flat clathrin coats (Sachse et al., 2002) and emanating tubules involved in recycling pathways that are described well on early endosomes, are also seen on late endosomes and, therefore, cannot be used to distinguish them. Comparable difficulties occur when distinguishing between endolysosomes and lysosomes, which have heterogeneous EM morphology. Hence, we have used a combination of criteria to identify late endocytic organelles, including the presence of ILVs and/or the presence of gold particles following a 4 h pulse/20 h chase fluid-phase uptake of colloidal gold stabilized with bovine serum albumin (BSA) and/or acid phosphatase labelling (Bright et al., 1997, 2016).

EM analysis of both thin (∼60 nm) and thick (250-300 nm) sections – the latter by electron tomography and 3D reconstruction – of VAMP7+VAMP8 double knockout cells, revealed three types of morphological change in the appearance of late endocytic organelles, when compared to those in control cells.

**Fig. 2.**
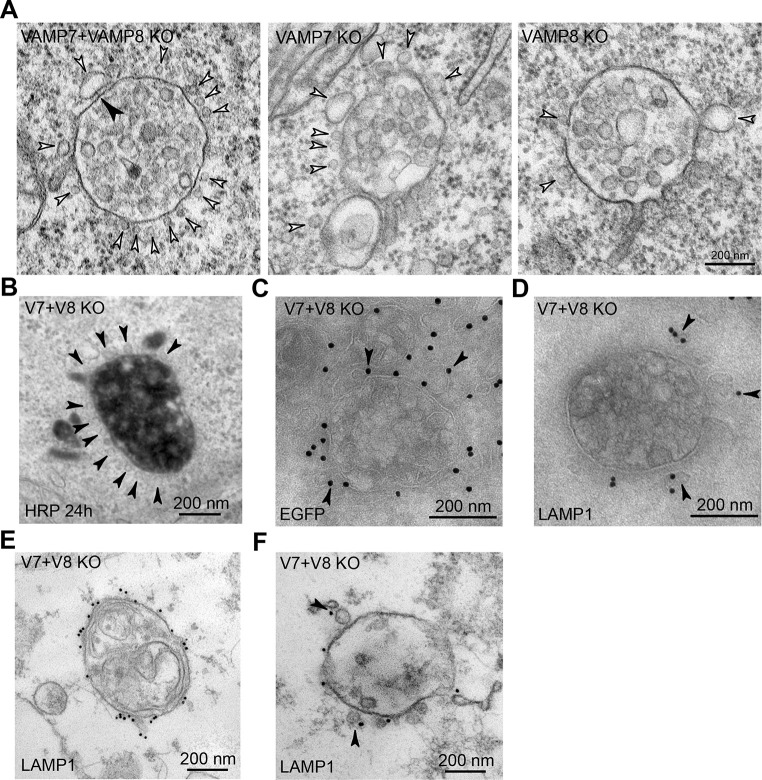
**Accumulation of small vesicles at MVBs in VAMP7+VAMP8 double knockout HeLa cells.** (A) Representative EM images of thin sections of chemically fixed VAMP7+VAMP8 double knockout HeLa cells and VAMP7 or VAMP8 single knockout cells showing the accumulation of small vesicles (white arrowheads) tethered to MVBs, especially in the double knockout cells. Mean vesicle diameter of the 14 vesicles shown was 37 nm (range of 26–108 nm). Filamentous tethers (black arrowhead) can also be observed. (B) High-power EM image of HRP-positive MVB showing that tethered vesicles – visible because of uranyl acetate ‘en bloc’ staining during processing (arrowheads) – are HRP-negative. (C) Representative image of anti-GFP labelled frozen section of an MVB from a VAMP7+VAMP8 double knockout cell after transfection with and expression of rat LAMP1/Lgp120-GFP for 16 h. Labelling shows tethered small vesicles (arrowheads). (D) Representative image of anti-LAMP1-labelled frozen section of an MVB from a VAMP7+VAMP8 double knockout cell after transfection with and expression of rat LAMP1/Lgp120-GFP for 16 h, showing labelling of tethered small vesicles (arrowheads). (E) Representative image of an endolysosome/lysosome from a VAMP7+VAMP8 double knockout cell. Pre-embedding labelling of endogenous LAMP1, showing labelling of the limiting membrane. (F) Representative image of an MVB from a VAMP7+VAMP8 double knockout cell. Pre-embedding labelling of endogenous LAMP1, showing labelling of tethered small vesicles (arrowheads).

**Fig. 3.**
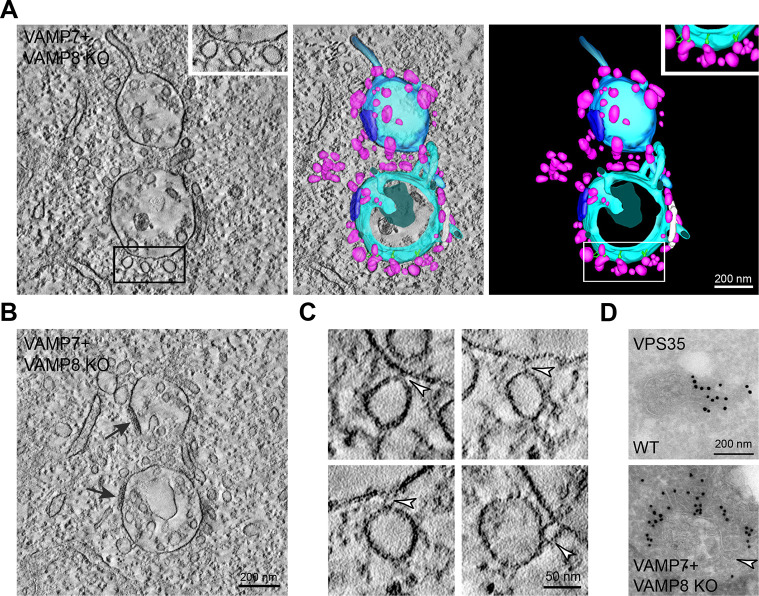
**Small vesicles tethered to MVBs in VAMP7+VAMP8 double knockout HeLa cells.** (A, left panel) EM image of a single tomographic slice (left panel) of a thick section of chemically fixed VAMP7+VAMP8 double knockout HeLa cell. (Middle and right panels) Reconstruction from the tomogram of a whole thick section showing small vesicles (pink in reconstruction) tethered to MVBs (reconstructed filamentous tethers shown in green); endosomes are shown reconstructed in turquoise and cyan. Boxed areas are shown magnified at top right of the respective image. Mean diameter of 86 reconstructed vesicles, 45 nm (range of 21–93 nm). Reconstruction images show the limiting membrane of MVBs and contiguous tubules (cyan), clathrin patches (dark blue) and tubular elements associated with but not contiguous with MVBs (white); see also Movie 1. (B) EM image of a single tomographic slice (different to that shown in A but from the same thick section), showing clathrin patches more clearly (arrows). (C) Four EM images at a higher magnification than A and B. Indicated are filamentous tethers (arrowheads) between individual vesicles and MVBs in VAMP7+VAMP8 double knockout HeLa cells, taken from individual tomographic slices derived from the thick section shown in A. (D) Representative images of immunolabelled VPS35 on tubules emanating from or close to an MVB in frozen sections of a WT (top) and a VAMP7+VAMP8 double knockout HeLa cell (bottom). An omega-shaped vesicle or organelle – probably in the process of fusing with the MVB – and devoid of VPS35 labelling, is indicated (arrowhead) in the bottom panel.

First, we observed the extensive accumulation of small vesicles tethered around MVBs but excluded from flat clathrin patches on these organelles (Figs 2A and 3A–C; Movie 1). Although we never saw accumulation of tethered vesicles around late endocytic compartments in control HeLa cells (data not shown), there was some accumulation in VAMP7 and VAMP8 single knockout cells, with the phenotype being more obvious in the former but not as clear as in the double knockout cells (Fig. 2A). The vesicle tethers were most clearly observed in tomogram reconstructions/videos and single tomographic slices of EM thick sections (Fig. 3A,C; Movie 1). The tethered small vesicles in the double knockout cells could not be identified by acid phosphatase staining (Fig. S2A), suggesting that they were not involved in trafficking newly synthesised acid hydrolases to late endocytic organelles. They were also not labelled following continuous uptake of horseradish peroxidase (HRP) for up to 24 h to label all endocytic compartments (Fig. 2B, Fig. S2B), implying that they did not derive from any part of the endocytic pathway. We, therefore, tested whether they were LAMP carriers that deliver newly synthesised LAMPs to late endosomes, using the criterion (established by Pols et al., 2013a) of whether they contained a transiently transfected tagged LAMP that had been expressed for 16 h. We transfected double knockout HeLa cells with GFP-tagged rat LAMP1 (also known as Lgp120) and, after 16 h, performed immuno-EM on frozen sections. We observed anti-GFP labelling of the limiting membrane of MVBs with some labelling also associated with tethered vesicles (Fig. 2C), consistent with them being LAMP carriers. In these cells, expressing GFP-tagged rat LAMP1, we also observed labelling of some vesicles tethered to MVBs with an antibody against the cytosolic domain of LAMP, although labelling of the limiting membrane of MVBs was very sparse (Fig. 2D). To provide further evidence that the tethered vesicles were LAMP carriers, we performed pre-embedding labelling of the VAMP7+VAMP8 double knockout cells with the antibody against the LAMP1 cytosolic domain. This labelled the limiting membrane of endolysosomal organelles containing internal membrane whorls (Fig. 2E) and some vesicles tethered to MVBs (Fig. 2F), but only sparsely labelled the limiting membrane of MVBs, except where ‘omega-shaped’ profiles were observed (Fig. S3). The omega-shaped profiles probably represent incomplete fusion of LAMP-positive lysosomes or large LAMP carriers with MVBs (see below).

Many of the MVBs with attached small vesicles observed by EM in the VAMP7+VAMP8 double knockout cells also had tubular elements associated with them (Fig. 3A). Some, which are closely apposed to but not contiguous with MVBs (white tubules in tomogram in Fig. 3A), are likely to be smooth endoplasmic reticulum (ER). These form membrane contact sites with MVBs and are implicated in diverse functions including regulation of endosomal positioning, signalling and lipid composition as well as fission of contiguous endosomal tubules during cargo sorting (Raiborg et al., 2015). In contrast, other tubules clearly extend from and are contiguous with the MVBs (Figs 2A and 3A). These tubules were shown by immuno-EM to be positive for the retromer component VPS35 (Fig. 3D), consistent with its requirement for recycling membrane proteins from endosomal compartments to the TGN and the plasma membrane (Burd and Cullen, 2014). Similarly, we also observed VPS35-positive tubules emanating from MVBs when using immuno-EM of control HeLa cells (Fig. 3D), and there was no obvious change in their frequency or disposition in the VAMP7+VAMP8 double knockout cells.

**Fig. 4.**
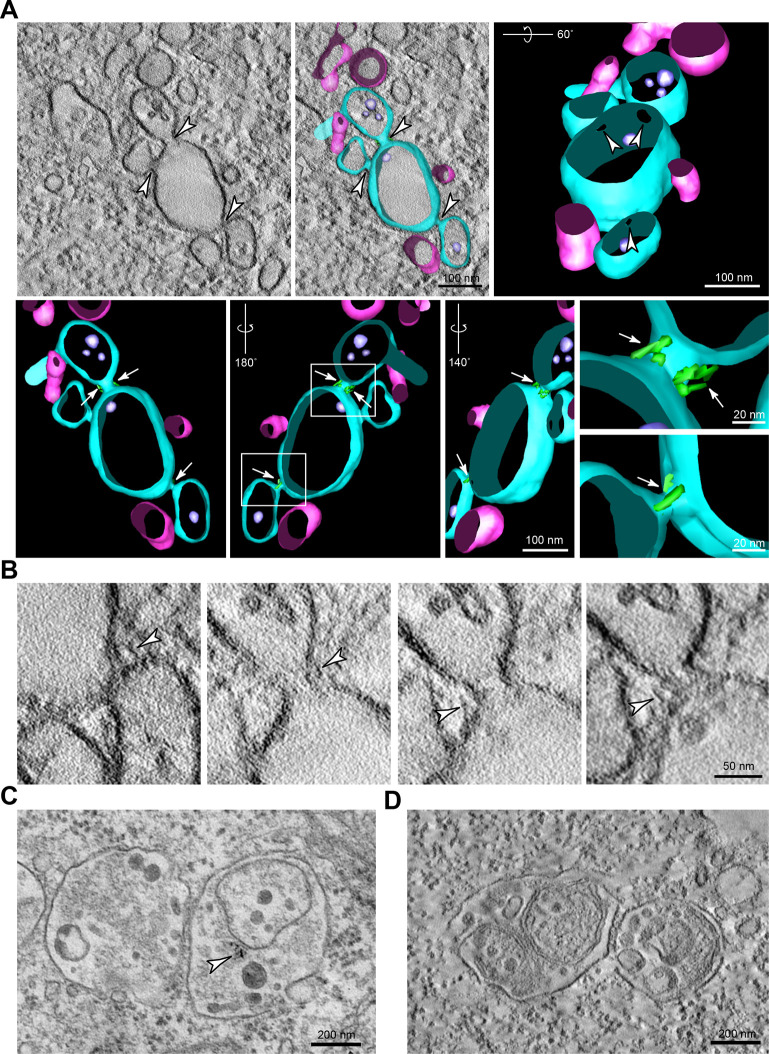
**Generation of hourglass profiles and tethering of late endocytic organelles in VAMP7+VAMP8 double knockout HeLa cells.** (A) Representative hourglass profiles in EM images of thick sections of chemically fixed cells. Individual tomographic slice (top left) and image reconstructions (remaining panels) from the tomogram of the whole thick section with enlargements; see also Movie 2. White arrowheads show the position of pores, white arrows indicate the position of tethers (reconstructed in green), although these are not visible in the single tomogram shown. Unconnected endosomal membranes are shown reconstructed in pink; endosomes connected by fusion pores are shown reconstructed in turquoise. (B) Four EM images at a higher magnification. Filamentous tethers (arrowheads) are shown at the edge of pores connecting hourglass bulbs of late endocytic organelles taken from individual tomographic slices of the thick section used in panel A. (C,D) EM image of a representative thin section (C) and individual tomographic slice from thick section (D) of chemically fixed cells. Shown are late endocytic organelles identified by an accumulation of colloidal gold (5 nm) stabilized with BSA (arrowhead) attached to each other by arrays of filamentous tethers.

The second morphological change we observed in the VAMP7+VAMP8 double knockout cells, was the presence of ‘hourglass’ and omega-shaped profiles of organelles that were likely to be in the process of fusion – although we cannot rule out fission. These organelles showed small pores linking their lumens, visible in image reconstructions from tomograms of whole thick sections, and a few filamentous tethers at the edge of the pores (Fig. 4A,B and Movie 2). The organelles appeared to be in the process of fusion and often had contiguous tubules likely to be involved in recycling membrane proteins, extending from them on the sides away from the sites of tethering and/or fusion (Fig. S4). Tethers close to the sites of constriction or necks of the hourglass profiles were most easily visible in tomogram reconstructions or videos and in single tomographic slices of EM thick sections, and were sometimes more apparent on one side of the pore (Fig. 4A,B and Movie 2).

The third morphological change we observed by EM in the VAMP7+VAMP8 double knockout cells was the occasional presence of late endocytic organelles joined to each other by arrays of ∼25 nm long filamentous tethers (Fig. 4C,D), a situation we have not observed in control HeLa cells. Observations of these larger arrays of tethers were rare and contrasted with observations of hourglass profiles in which only a few tethers were present, close to the pores between organelles (Fig. 4A,B).

### Depletion of CHMP6 causes extensive tethering of MVBs to each other and to swollen endolysosomes

To better understand the relationship between tethering and pore formation, we sought another cell treatment or manipulation that would result in more-extensive organelle tethering and allow us to search for pores linking the lumen of the tethered organelles. Although occasional images of tethered late endocytic organelles in response to different treatments have previously been reported (Futter et al., 1996; Bright et al., 1997; Row et al., 2006), we decided to re-visit in greater detail the morphology and attachment of clustered late endocytic organelles that occurs reproducibly after depletion of the ESCRT-III component CHMP6 (Parkinson et al., 2015). In HeLa cells, we have found previously that depletion of CHMP6 mediated by small interfering RNA (siRNA) neither prevented the formation of MVBs nor the degradation of endocytosed ubiquitylated MHC class I molecules through lysosomal proteases (Parkinson et al., 2015). However, depletion of CHMP6 did cause disruption of the autophagy pathway – which was also observed following depletion of other individual ESCRT proteins (Rusten and Stenmark, 2009) – as well as a profound alteration in the morphology of compartments on the endocytic pathway (Parkinson et al., 2015 and Fig. S5A,B). The most notable change was the development of a swollen endolysosomal phenotype, often with associated clusters of MVBs, apparent from ∼24 h after siRNA treatment. This morphological phenotype correlated with the extent of CHMP6 depletion and was rescued by expression of siRNA-resistant CHMP6 (Parkinson et al., 2015).

**Fig. 5.**
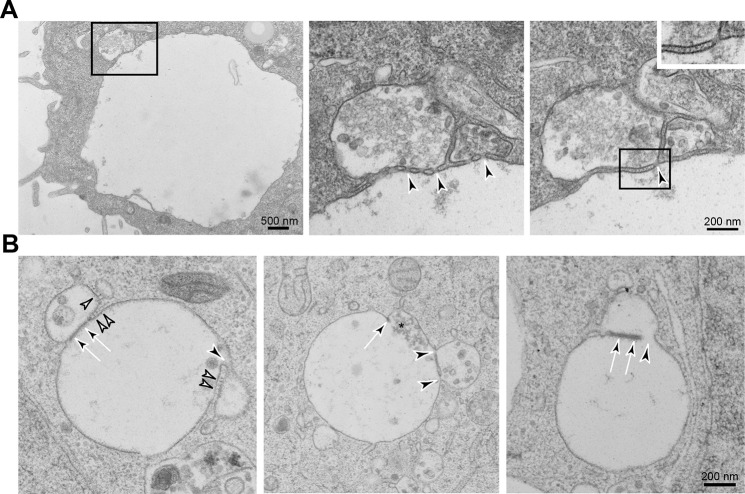
**Tethering and pore formation between the limiting membranes of MVBs and swollen endolysosomes in CHMP6-depleted HeLa cells.** (A) Electron micrograph of a thin section of a chemically fixed CHMP6-depleted HeLa cell showing clustered MVBs tethered to each other and a swollen endolysosome. Magnifications of serial sections of the boxed area are shown in the two panels on the right. The boxed area in the right image is shown further enlarged (top right), showing filamentous tethers. Pores (arrowheads) are clearly visible in tethered regions between MVBs and the swollen endolysosome. (B) High-pressure frozen thin sections of CHMP6-depleted HeLa cells reveal filamentous tethers (white arrowheads), closely apposed regions of membrane (arrows) and pores (black arrowheads), between tethered MVBs and enlarged endolysosomes. The asterisk in the middle panel indicates an MVB that has probably discharged contents (*) into a swollen endolysosome through an expanded fusion pore but has retained a remnant of tethered membrane (arrow).

EM of thin sections of conventionally fixed HeLa cells, in which CHMP6 had been depleted using siRNA, confirmed the presence of swollen endolysosomal compartments often closely associated with MVBs (Fig. 5A). Immuno-EM revealed that the swollen compartments and MVBs were positive for LAMP1 and ubiquitin, and that the MVBs were positive for ciMPR (data not shown). EM cytochemistry confirmed that the swollen endolysosomes and, often but not always, the associated MVBs were positive for acid phosphatase (data not shown), consistent with the fluorescence microscopy results, in which the swollen compartments were labelled with antibodies against cathepsin D and were Magic Red positive (Fig. S5A). Thus, the swollen compartments were proteolytically active endolysosomes.

**Fig. 6.**
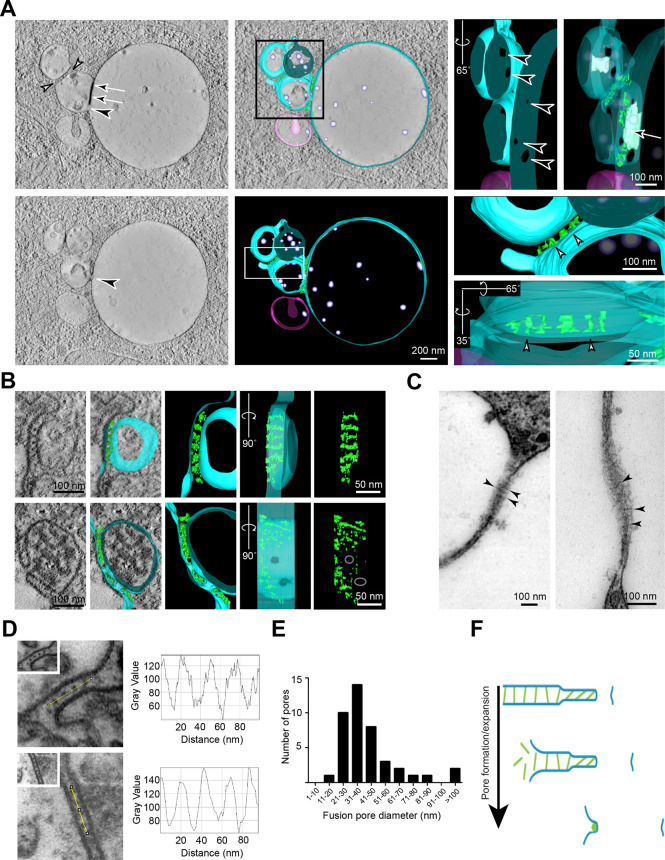
**Fusion pores and filamentous tethers linking MVBs and swollen endolysosomes in CHMP6-depleted HeLa cells.** (A) Tomographic slices from two image planes (top left, bottom left) within a thick section of a high pressure frozen CHMP6-depleted HeLa cell reveal multiple fusion pores (black arrowheads) connecting the swollen endolysosome and an adjacent MVB in addition to closely apposed tethered regions (arrows) and filamentous tethered regions (white arrowheads). Reconstruction of the whole thick section and magnifications of the boxed areas reveal pores and tethers (green) when shown at high degrees of rotation and/or translucent membranes. Endosomes connected via fusion pores shown in turqoise, unconnected endosome shown in pink. See also Movie 3. (B) Images of single tomographic slices and reconstructions from tomograms of thick sections of chemically fixed CHMP6-depleted cells showing examples of tethered late endocytic organelles. A 90° rotation of the 3D reconstruction of filamentous tethers (green) reveals an ordered array between organelles lacking a fusion pore; periodicity is disrupted in the immediate vicinity of fusion pores. Endosomes are shown reconstructed in turquoise. (C) Examples showing the periodicity of filamentous tethers (arrowheads) between late endocytic organelles, observed in electron micrographs of conventional thin sections of chemically fixed CHMP6-depleted cells, whose tethered region was sectioned obliquely. (D) (Left) Thin sections of chemically fixed CHMP6-depleted HeLa cells. Magnified views of the boxed areas in each panel show filamentous tethering regions observed between clustered late endocytic organelles. In the top left image, tannic acid was used during processing as a mordant to augment heavy metal staining. (Right) Pixel intensity line scans of 100 nm (yellow lines connecting dots in left hand panels) along these tethered arrays reveal the periodicity of the tethers. (E) Bar graph showing the number of pores over a range (1– >100 nm) of fusion pore diameters (42 measurements) between tethered late endocytic organelles in thin sections of high-pressure frozen CHMP6-depleted cells. (F) Schematic cross-section diagram illustrating pore formation at the edge of closely apposed membranes/filamentous tether arrays. Tethers are depicted in green; organelle-limiting membranes are depicted in blue.

In EM thin sections, we also observed that the MVBs were tethered to each other as well as to the swollen endolysosomes by ordered arrays of ∼25 nm long filamentous tethers and that small pores, linking the organelle lumens, were often seen associated with the tethered regions (Fig. 5A,B). The individual tethers between MVBs as well as those linking MVBs to swollen endolysosomes observed in thin and thick sections (Figs 5A,B and 6A-D) were very similar in appearance to those described above, i.e. following double knockout of VAMP7 and VAMP8 (Fig. 4C,D) and in HEp-2 cells following uptake of HRP-EGF (Futter et al., 1996). However, the tether arrays were much more extensive. Tethers and pores were not an artefact of conventional chemical fixation (Murk et al., 2003) since they were also observed by EM of thin or thick sections of CHMP6-depleted HeLa cells prepared by high pressure freezing (HPF) with freeze substitution (Figs 5B and 6A and Movie 3). By using this technique, the arrays of filamentous tethers linking MVBs and swollen endolysosomes were observed more clearly. Moreover, they often emanated from closely apposed, flattened areas of limiting membranes of the organelles, into which the electron-dense OsO_4_ staining had penetrated (Fig. 6A), similar to the flattened plaque-like areas previously described in HEp-2 cells following uptake of HRP-EGF (Futter et al., 1996). The length of filamentous tethers in HPF thin sections of CHMP6-depleted HeLa cells was 22±0.8 nm (*n*=50) and the distance between the limiting membranes in the closely apposed, flattened areas was 11±0.7 nm (*n*=29). The distance between the mid-points, i.e. periodicity, of the filamentous tethers within tether arrays was 23±0.6 nm (*n*=12), measured by line scanning of chemically fixed thin sections (Fig. 6D). This was consistent with the periodicity of tethers from thick sections of HPF samples observed after image reconstruction (see example in Fig. 6A, Movie 3). The tethers were also clearly observed in occasional oblique thin sections of apposed membranes in chemically fixed samples (Fig. 6C).

The multi-subunit HOPS complex has been proposed to function as a physical tether between late endocytic organelles and yeast HOPS, structurally characterised as a flexible elongated protein complex of ∼30 nm that may exist in a closed or open conformation (Bröcker et al., 2012; Chou et al., 2016; Lurick et al., 2018), and shown to act as a tether in a proteoliposome system (Hickey and Wickner, 2010). By using confocal immunofluorescence microscopy we observed that, in CHMP6-depleted HeLa cells, the human HOPS protein vacuolar protein sorting 18 (VPS18), which is also a component of the class C vacuole/endosome tethering (CORVET) complex (Balderhaar and Ungermann, 2013), is associated with the membranes of swollen and clustered LAMP1-positive late endocytic compartments (Fig. S6A). This was confirmed by immuno-EM using pre-embedding labelling (Fig. S6B). However, using these techniques, we were unable to see antibody labelling of the tethers themselves, possibly because we were unable to achieve sufficiently high labelling density due to poor antibody access to epitopes embedded in the tightly packed tether arrays – a situation we were unable to improve by overexpressing tagged-VPS18 (data not shown). Nevertheless, the development of the swollen endolysosomal phenotype in CHMP6-depleted HeLa cells was dependent on the presence of the HOPS component VPS18, because depletion of this protein before or during, but not after, depletion of CHMP6 prevented the development of the phenotype and the effect of VPS18 depletion was rescued by expressing siRNA-resistant VPS18 (Fig. S6C).

### Pores between MVBs and swollen endolysosomes are observed near the edge of filamentous tether arrays

In the high-resolution EM images of thin sections of HeLa cells in which CHMP6 was depleted, we often observed small membrane-bound pores in the filamentous tethered regions (Figs 5A,B and 6B), thus creating a continuum between the lumen of MVBs and swollen endolysosomes, and between tethered MVBs. The pores were also observed in thick sections cut from HPF samples and examined by using tomography and 3D reconstruction. In one reconstruction shown (Fig. 6A, Movie 3), we observed that the three pores between an MVB and a swollen endolysosome were located towards the edge of the region of tethering, which was also true in other cases (Fig. 6B and other examples not shown). Our impression that pores were preferentially formed near the edge of tethered regions was reinforced by further examination of HPF thin sections (e.g. Fig. 5B). Observation of larger fusion profiles often revealed a plaque or button of residual tether(s) on one side of the fusion profile (Fig. 5B). Interestingly, quantitative analysis of 42 pores observed in HPF thin sections showed a diameter range of 19–148 nm (with 60% being <40 nm; Fig. 6E), suggesting that, when small pores are formed, they can expand (see schematic in Fig. 6F). Both in HPF thin sections and in reconstructions of tethered regions from chemically fixed thick sections, we observed disruption of the regular tether array close to the pore (see Fig. 5B for HPF thin section; Fig. 6B for chemically fixed thick sections), consistent with pore expansion being coupled to the release of filamentous tethers.

### Lipidated R-SNARE YKT6 compensates for the absence of VAMP7 and VAMP8 in the endocytic delivery to lysosomes

**Fig. 7.**
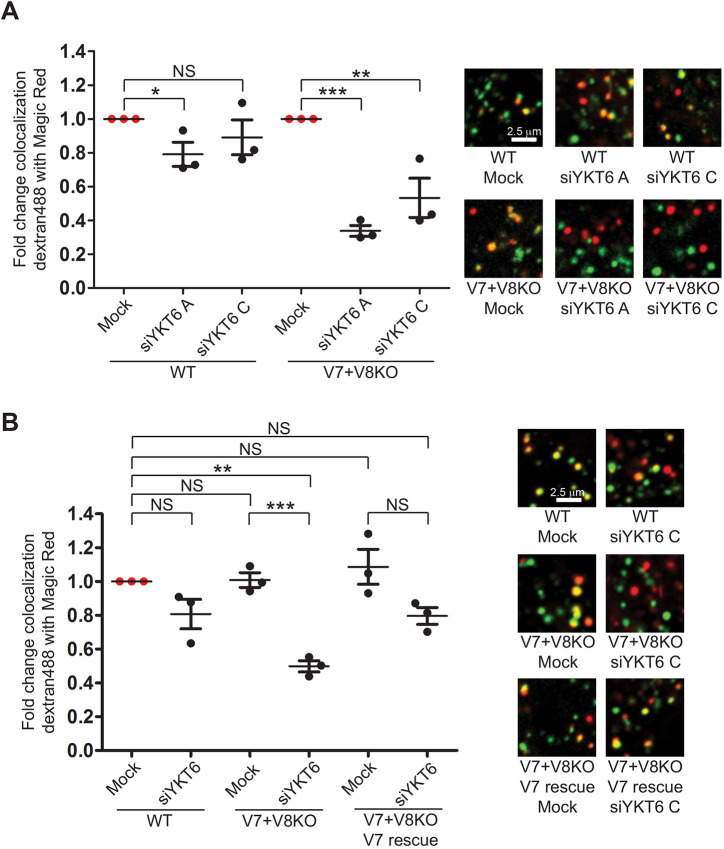
**Effect of YKT6 depletion on endocytic delivery to lysosomes in VAMP7+VAMP8 double knockout cells.** (A) Colocalization (Manders’ coefficient) of fluorescent dextran (loaded for 2 h, chased for 1 h) with hydrolysed cathepsin B Magic Red substrate in wild-type (WT) and VAMP7+VAMP8 double knockout (V7+V8 KO) HeLa cells, in which YKT6 was depleted using siRNA oligonucleotides siYKT6A or siYKT6C. For the three experiments shown, YKT6 depletion was 94.2±1.8% (mean±s.e.m.) with siYKT6A and 83.3±9.0% with siYKT6A in wild-type cells and 94.6±0.4% with siYKT6A and 79.5±11.3% with siYKT6C in double knockout cells; see also Fig. S7D. (B) The effect of expressing HA-VAMP7 on colocalization (Manders’ coefficient) of fluorescent dextran (loaded for 2 h, chased for 1 h) with hydrolysed cathepsin B Magic Red substrate in VAMP7+VAMP8 double knockout HeLa cells in which YKT6 was depleted using siRNA siYKT6C. For the three experiments shown, YKT6 depletion with siYKT6C was 69.3±5.4% (mean±s.e.m.) in wild-type cells, 66.5±7.1% in double knockout cells, and 58.9±6.1% in double knockout cells expressing HA-VAMP7; see also Fig. S7F,G. Data in A and B are the means±s.e.m of three independent experiments with five fields each, ≥30 cells total per condition. ***P<0.001, **P<0.01 (two-tailed unpaired *t*-test), NS, not significant. Representative confocal microscopy images are shown on the right of A and B.

As described above, in contrast to the morphological phenotype exemplified by the altered distribution of LAMP1 labelling, we observed no effect on endocytic uptake and delivery of fluorescent dextran to Magic Red-positive endolysosomes when knocking out VAMP7 and VAMP8 individually or together (Fig. 1C and Fig. S1F). Moreover, we observed neither increased expression of the other longin-domain-containing R-SNAREs YKT6 and SEC22b, nor of the R-SNAREs VAMP2 and VAMP3 in VAMP7+VAMP8 double knockout HeLa cells (Fig. S7A). Nevertheless, we sought to find out whether YKT6 has a compensatory role, as its yeast orthologue Ykt6p can substitute for the VAMP7 orthologue R-SNARE Nyv1p in yeast vacuole fusion (Thorngren et al., 2004) and VAMP7 can substitute for YKT6 in the fusion of autophagosomes with lysosomes in *Drosophila* (Takáts et al., 2018). There was no obvious alteration in intracellular distribution of YKT6 within VAMP7+VAMP8 double knockout HeLa cells, when observed using confocal immunofluorescence microscopy (Fig. S7B,C). Nevertheless, depletion of YKT6 with either of two independent siRNAs (Fig. S7D) significantly reduced delivery of fluorescent dextran to Magic Red-positive endolysosomes in VAMP7+VAMP8 double knockout HeLa cells (Fig. 7A), without affecting endocytic uptake (Fig. S7E). The fact that overexpressed mammalian YKT6 does not target to membranes correctly and remains cytosolic makes rescue experiments with this SNARE challenging (Gordon et al., 2017). However, in rescue experiments, we found that the effect of YKT6 depletion in double knockout cells was significantly reduced when haemagglutinin A (HA)-tagged VAMP7 had been transiently overexpressed (Fig. 7B, Fig. S7F,G). In addition, the more-extensive depletion of YKT6 inhibited endocytic delivery to lysosomes in both control wild-type and VAMP7+VAMP8 double knockout cells (data not shown).

## DISCUSSION

Here, we explored two conditions under which increased tethering was observed between late endocytic organelles in HeLa cells, enabling us to gain insights into the relationship between tethers and membrane pore formation. Knockout of VAMP7 or double knockout of VAMP7 and VAMP8 resulted in a relatively mild phenotype, in which there was an alteration in the morphology of late endocytic organelles but no apparent defect in delivering endocytosed fluorescent dextran to acid hydrolase-active endolysosomes. Our EM examination of VAMP7+VAMP8 double knockout cells showed the accumulation of LAMP carriers (Pols et al., 2013a) tethered to MVBs, hourglass profiles of attached organelles with small pores linking their lumens and tethered late endocytic organelles. Taken together, these morphological changes are consistent with the increase in size of LAMP1-positive puncta observed by immunofluorescence microscopy.

In the VAMP7+VAMP8 double knockout HeLa cells, the necks of the hourglass profiles observed by EM were associated with a few (∼25 nm-long) filamentous tethers, attached to each ‘bulb’ of the hourglass on the cytosolic side of the membrane. Occasionally, arrays of filamentous tethers were observed linking late endocytic organelles, a phenomenon seen much more often when the endocytic pathway was more severely disrupted after depletion of the ESCRT-III protein CHMP6 in HeLa cells. Additionally, we observed small pores at the edges of the arrays of filamentous tethers linking late endocytic organelles in CHMP6-depleted HeLa cells. In both VAMP7+VAMP8 double knockout and CHMP6-depleted cells, the morphological phenotypes observed by EM suggested that we had generated conditions of frustrated fusion, during which late endocytic organelles were tethered, pores could form, but only some had expanded to enable full fusion. This has some similarities to frustrated exocytosis produced experimentally in *Paramecium* when secretory granules fuse with the plasma membrane, do not release all contents but can then be detached and, subsequently, re-attach (Klauke and Plattner, 2000). Just as frustrated exocytosis has provided insights into the normal secretory process in *Paramecium*, we suggest that our data provides insights into the membrane fusion events required for delivery of endocytosed cargo from late endosomes to lysosomes in non-manipulated mammalian cells.

Our observations are consistent with a model in which delivery of endocytosed cargo to lysosomal proteases is the result of late endocytic organelles being bound by filamentous tethers that are likely to be composed of HOPS proteins. Tethering leads to very close apposition of the membranes before formation of small (∼10–50 nm) pores at the edge of the tethered  and apposed regions, which can expand leading to full fusion. The trans-SNARE complexes required for pore formation and nascent pore expansion (Bao et al., 2018), are also likely to be involved in the close apposition of the membranes after initial tethering. Interestingly, studies with yeast vacuoles suggest that tethers of the HOPS complex play an essential mechanical role in the terminal stages of phospholipid bilayer fusion (D'Agostino et al., 2017), in addition to their tethering function and involvement in regulating trans-SNARE complex formation (Baker et al., 2015; Schwartz et al., 2017; Song et al., 2020). Pore expansion is coupled to the release of filamentous tethers. This release could be achieved by activation of a GTPase-activating protein (GAP) for Rab7 or Arl8B – the small GTPases required to recruit HOPS to membranes (van der Kant et al., 2015; Khatter et al., 2015). Moreover, it is interesting that TBC1D2 (also called Armus) – one of three Rab7 GAPs implicated in delivery of endosomal cargo to lysosomes (reviewed by Stroupe, 2018) – is activated by the kinase LRRK1, has been shown to be required for such delivery and binds to the open form of VAMP7 in a newly formed trans-SNARE complex (Toyofuku et al., 2015; Wang et al., 2018).

Merging of multiple adjacent fusion pores has previously been suggested as a means to complete vertex ring fusion of yeast vacuoles to achieve mixing of luminal content (Wang et al., 2002). However, it should be noticed that, in a study of vacuole fusion in live wild-type yeast cells, 6.1% of all fusion events observed did not occur by vertex ring fusion but were more likely explained by pore dilation and/or expansion (Mattie et al., 2017). In our experiments using cultured HeLa cells, even following depletion of CHMP6, we never observed a ring of pores all the way around the tethered/apposed membranes that, when merged, released boundary membrane into the lumen of the resultant fused organelle, as occurs during vertex ring fusion of yeast vacuoles. If they fail to expand, small pores of the type seen in our experiments can also explain kiss-and-run events between late endosomes and lysosomes in non-manipulated mammalian cells when some content is exchanged – the membranes re-seal and the organelles become untethered. Opening and closing of fusion pores to allow kiss-and-run exocytosis as well as expansion of fusion pores following docking of secretory organelles at the plasma membrane have been well described in mammalian cells (Takahashi et al., 2002; Jaiswal et al., 2004; Staal et al., 2004; Alabi and Tsien, 2013).

In the VAMP7+VAMP8 double knockout HeLa cells, the observation that there is no reduction in delivery of endocytosed fluorescent dextran to cathepsin-active endolysosomes implies the action of a compensatory R-SNARE, which our experiments suggest is YKT6. To date, we have studied neither the delivery of other cargoes to the acid hydrolase-active endolysosomes, nor the delivery of newly synthesised cathepsin. It is, however, clear from the use of Magic Red that endolysosomes in the knockout cells contain sufficient active cathepsin B to yield a good fluorescence signal – even in experiments in which YKT6 had been depleted. YKT6 is a longin domain-containing R-SNARE that does not contain a trans-membrane domain but is both farnesylated and palmitoylated, and exists in membrane-bound and cytosolic pools. Although both lipid modifications are essential for stable association of YKT6 with the membrane, palmitoylation-dependent cycling on and off membranes is reversible, whereas farnesylation is essentially irreversible and regulates its fusion activity (Wen et al., 2010). In different species, YKT6 is both a versatile and promiscuous SNARE that can function at many steps in intracellular membrane traffic pathways. These include ER-to-Golgi, intra-Golgi, endosome-to-Golgi and Golgi-to-plasma membrane transport; traffic to the yeast vacuole; vacuole fusion; endosome fusion with the plasma membrane (all summarised by Gordon et al., 2017) and fusion between autophagosomes and lysosomes or vacuoles (Takáts et al., 2018; Bas et al., 2018; Gao et al., 2018; Matsui et al., 2018). In ER-to-Golgi transport YKT6 functions redundantly with SEC22b (Liu and Barlowe, 2002; Gordon et al., 2017) and Ykt6p in yeast can substitute for Nyv1p in vacuole fusion, a property suggested to explain the non-fragmented morphology of vacuoles in *nyv1Δ* strains (Thorngren et al., 2004). Our data are consistent with YKT6 being a compensatory R-SNARE for endolysosomal fusion events when VAMP7 and VAMP8 are both absent. The observation that more-extensive depletion of YKT6 also affects endocytic delivery to lysosomal hydrolases in wild-type cells, suggests that, normally, it also has another direct or indirect role in endolysosomal fusions. In this regard, it is worth noting that Ykt6p in yeast has been implicated to regulate the palmitoylation of the armadillo-repeat protein Vac8p that is required for efficient vacuole fusion (Dietrich et al., 2005).

## MATERIALS AND METHODS

### Reagents

Roswell Park Memorial Institute medium (RPMI 1640), fetal calf serum (FCS), Dulbecco's phosphate buffered saline (PBS), cytidine 5′-monophosphate disodium salt, cerium(III) chloride heptahydrate, beta-glycerophosphate disodium salt hydrate, glutamine and penicillin-streptomycin, horseradish peroxidase (HRP, Type VI) and 3,3′-diaminobenzidine (DAB) were from Sigma-Aldrich (Poole, UK). Tris-buffered saline (TBS) was 0.82% NaCl, 100 mM Tris pH 8.0 and TBS-T was TBS containing 0.1% TWEEN 20 from NBS Biologicals (Huntingdon, UK). Dried skimmed milk powder (Marvel) was from Premier Foods (St Albans, UK). Magic Red substrate for cathepsin B assay was from ImmunoChemistry Technologies (Bloomington, MN). Bafilomycin A1 was from Alfa Aesar (Heysham, UK). Hoechst 33342, Geneticin (G418), LysoTracker Green and Dextran (10 000 MW, anionic, fixable) conjugated to Alexa Fluor 488 were from Life Technologies/ThermoFisher Scientific (Waltham, MA). Antibodies were: mouse monoclonal anti- cation-independent mannose-6-phosphate receptor (clone 2G11), rabbit polyclonal anti-LAMP1 (to a cytosolic tail epitope, ab24170), rabbit polyclonal anti-GFP (ab6556) and rabbit anti-mouse IgG (ab6709) (all from Abcam, Cambridge, UK), rabbit polyclonal anti-LC3B (NB 100-2220) (Novus Biologicals, Bio-Techne Ltd, Abingdon, UK), mouse anti-VPS35 (clone B-5; Santa Cruz Biotechnology, Dallas, TX), mouse monoclonal anti-EEA1 (clone 14/EEA1) and mouse monoclonal anti-LAMP1 (clone H4A3) (both from BD Biosciences, San Jose, CA), mouse monoclonal anti-ubiquitin (clone FK2; Enzo Life Sciences, Farmingdale, NY), rabbit anti-cathepsin D (219361) and rabbit anti-actin (A2066) (both from Sigma-Aldrich (Poole, UK), mouse monoclonal anti-VAMP2 (clone 541405; R&D Systems, Minneapolis, MN) and rabbit anti-SEC22B (Synaptic Systems, Göttingen, Germany). Rabbit anti-VAMP3, mouse anti-VAMP7 and rabbit anti-VAMP8 were prepared and validated as previously described (Gordon et al., 2010), as were the affinity-purified rabbit polyclonal antibodies against human VPS18 (aa 40-394) and human VPS33A (Graham et al., 2013; Wartosch et al., 2015). Affinity-purified rabbit anti-YKT6 was a gift from Dr Jesse C. Hay (University of Michigan) to A.A.P. as previously reported (Gordon et al., 2010) and was raised against the peptide DGHLSRYQNPREADPMSKC, and was conjugated to keyhole limpet hemocyanin as previously described (Hasegawa et al., 2003). Donkey anti-mouse Ig: Alexa Fluor 594 (A21203), donkey anti-rabbit Ig: Alexa Fluor 594 (A21207), donkey anti-mouse Ig: Alexa Fluor 488 (A32766) and donkey anti-mouse Ig: Alexa Fluor 647 (A31571) were from ThermoFisher Scientific (Waltham, MA). Rabbit anti-mouse: HRP (A9044) and mouse anti-rabbit: HRP (A1949) were from Sigma-Aldrich (Poole, UK). Goat anti-mouse: IRDye 680RD (926-68070) and goat anti-rabbit: IRdye 680 (926-32221) were from LI-COR Biosciences (Lincoln, NE). Protein A–gold (PAG) conjugates were from the Department of Cell Biology, University of Utrecht. Fab fragments of goat anti-rabbit IgG conjugated to 1.4 nm nanogold particles and GoldEnhance™ EM plus (2004) were from Nanoprobes (Yaphank, NY)

### Cell culture and delivery of endocytosed dextran to endolysosomes/lysosomes

HeLa M cells (a gift from Dr P. J. Lehner, University of Cambridge; described in Hewitt et al., 2002) were verified using the Eurofins cell line authentication service and were negative for mycoplasma contamination [routinely tested for the presence of mycoplasma contamination using MycoAlert™ Mycoplasma Detection Kit (LT07-318) from Lonza Bioscience Blackley, UK, and treated with mycoplasma removing agent BM-Cyclin (10 799 050 001) from Roche Diagnostics, Burgess Hill, UK]. The cells were cultured in RPMI supplemented with 10% (v/v) FCS, 2 mM glutamine, 100 U/ml penicillin and 100 μg/ml streptomycin. The delivery of endocytosed dextran to Magic Red-positive endolysosomes/lysosomes in which Cresyl Violet was retained as a consequence of the lumenal acidic environment (Ostrowski et al., 2016), was measured as previously described (Wartosch et al., 2015), using quantitative confocal microscopy of live cells. In brief, cells were first loaded for 2 h with 1 mg/ml dextran conjugated to Alexa Fluor 488 in RPMI followed by a chase for 1 h in dextran-free RPMI. Cells were then rinsed with PBS and incubated with Magic Red–cathepsin B substrate in CO_2_-independent medium (ThermoFisher Scientific, #18045-054) for 5 min before examination in a Zeiss LSM 780 confocal microscope. Colocalization of the dextran fluorescence and Magic Red signal was calculated as previously described (Wartosch et al., 2015). For analysis of total cellular uptake of dextran–Alexa Fluor 488 (Wartosch et al., 2015), cells were loaded as for the experiment measuring delivery to endolysosomes/lysosomes but then subjected to FACS analysis by using a five-laser LSR Fortessa (BD Biosciences) with 30,000 cells per condition analysed with FLOWJO software.

### CRISPR/Cas9 knockout of VAMP7 and VAMP8

CRISPR gRNAs were designed using an online CRISPR design tool. Together with *Streptococcus pyogenes* Cas9 they were expressed from the pX330 plasmid (Addgene #42230, deposited by Fen Zhang; Cong et al., 2013). Complementary oligonucleotides designed to form BbsI overhang-containing duplexes were from Sigma-Aldrich and for exon 2 of *VAMP7*: 5′-CACCGAACAAACTAACGTACTCACA-3′ and 5′-AAACTGTGAGTACGTTAGTTTGTTC-3′; exon2 of *VAMP8*: 5′-CACCGTGGAGGAAATGATCGTGTG-3′ and 5′-AAACCACACGATCATTTCCTCCAC-3′.

For each gene or exon, sense and antisense oligonucleotides were phosphorylated together using T4 polynucleotide kinase (#M0201S, NEB, Ipswich, MA) according to manufacturer's instructions. They were then duplexed by denaturing at 95°C for 5 min before decreasing the temperature to below 30°C at a rate of −0.5°C/min. Plasmid PX330 was digested with BbsI (#R0539, NEB) and ligated with the relevant oligonucleotide duplex using T4 DNA ligase (#M0202, NEB) according to manufacturer's instructions. Cells were co-transfected with the PX330-gRNA construct and pIRES-GFP-Puro (Addgene #45567, a gift from Michael McVoy) in a ratio of 1:5, using lipofectamine 2000 (#11668027, ThermoFisher, Waltham, MA) according to manufacturer's instructions for 100 mm dishes. Cells were trypsinised 48 h after transfection, washed with PBS and resuspended at a concentration of 10^6^/ml in PBS. Sorting was carried out using a BD Influx™ cell sorter. Single GFP-positive cells, gated at a fluorescent intensity above all events seen in a control population, were collected and seeded into a 1:1 mix of fresh RPMI with 24 h-conditioned RPMI in 96-well Cellstar^®^ cell culture microplates (#655180, Greiner Bio-One) for expansion and to generate individual clones.

### Genomic DNA isolation and sequencing

Genomic DNA was isolated from ∼1×10^6^ cells using the High Pure PCR template preparation kit (Roche Diagnostics) according to the manufacturer's instructions. The region around the CRISPR/Cas9 lesion was amplified using primers 5′-CAGGGGGACCACTATCCTTG-3′ for VAMP7 and 5′-AGTCTGCCTGAGGCCTTACC-3′ for VAMP8. PCR products were cloned into pCR-Blunt vector (ThermoFisher), transformed into *E. coli* and resulting colonies were amplified and screened by diagnostic EcoRI restriction digest for the presence of an integration event. Recovered plasmids from positive colonies were analysed by Sanger sequencing with the same primers used in amplification of the region.

### siRNA treatment, protein depletion and rescue experiments

All siRNAs were from Dharmacon/Thermo Scientific. The CHMP6 siRNA double transfection, knockdown protocol was carried out as described previously (Parkinson et al., 2015), using the siGENOME SMART pool M-005060-01. Depletion of human VPS18 was carried out by transfection of *VPS18* siRNA 48 h before, at the same time or 48 h after each CHMP6 knockdown, using the VPS18 SMART pool L-013178-00. Individual *VPS18* siRNA oligonucleotides #3 and #4 were 5′-GAGCUACUUUGAGGAGAUU-3′ and 5′-GACGUAAGGAUGACGCAAA-3′, respectively. For the VPS18 rescue experiment, GFP- tagged mouseVPS18, containing four mutations in the region of sequence identity with oligonucleotide #4 siRNA was cloned into pIRESneo2 and the resulting plasmid used to transfect HeLa cells with TransIT-HeLa Monster^®^ (Mirus, Madison, WI), followed by antibiotic selection of stably expressing cells. Subsequent treatment of these cells with oligonucleotide #4 or the SMART pool siRNA for VPS18 was as described above. VPS33A depletion was achieved with a double transfection protocol, i.e. two transfections separated by 48 h, with the SMARTpool L–013330–01, using Oligofectamine (Life Technologies) according to the manufacturer's protocol. Depletion of YKT6 was achieved by following a single transfection protocol using Oligofectamine and two, previously validated (Gordon et al., 2010), custom siRNA oligonucleotides with the sequences siRNA oligo A, 5′-GCUCAAAGCCGCAUACGAU-3′ and siRNA oligo C, 5′-AUACCAGAACCCACGAGAA-3′.

VAMP7+VAMP8 double knockout cells stably expressing HA-VAMP7 were generated using the pLXIN retroviral system as previously described (Gordon et al., 2009). Successfully transduced cells of mixed expression levels were selected by culture in RPMI with 0.5 mg/ml Geneticin (#10131027, Life Technologies). In all experiments where mock knockdown was used as a control, transfection under the same conditions with non-targeting (NT) control D–001810–01 (5′-UGGUUUACAUGUCGACUAA-3′) was used. In all RNA interference (RNAi) experiments, cells were examined 48 h after the final knockdown transfection, unless otherwise stated and protein depletion was assessed by immunoblotting as described previously (Parkinson et al., 2015) and was always >85% for CHMP6, VPS18 and VPS33A. For RNAi of YKT6, protein depletion is stated in legend to Fig. 7.

### Immunoblotting

Cells were lysed in SDS buffer [2% SDS, 50 mM NaCl, 50 mM Tris-HCl pH 7.4, 1× cOmplete™ protease inhibitor cocktail (Roche Diagnostics)] before boiling in TruPAGE™ LDS sample buffer (Sigma-Aldrich) supplemented with 0.1 M DTT for 10 min. SDS-PAGE was performed on hand-cast or TruPAGE™ SDS-polyacrylamide gels with TruPAGE™ TEA-Tricine SDS running buffer (Sigma) using the omniPAGE Mini Vertical Protein Electrophoresis System (Cleaver Scientific, Rugby, UK). Following transfer of proteins using a Mini-PROTEAN 2-D Electrophoresis Cell (Bio-Rad, Hercules, CA) to Immobilon^®^-P PVDF membranes (Merck-Millipore, Watford, UK) for chemiluminescence detection or for fluorescence detection, membranes were blocked with 5% dried skimmed milk powder in TBS-T. Incubations with primary antibodies (1:1000 dilution, except anti-SEC22B, 1:500, and anti-actin, 1:6000) were carried out overnight at 4°C, with appropriate horseradish peroxidase- or IRDye-conjugated secondary antibodies (1:1000 dilution) applied subsequently for 1 h at room temperature. Fluorescent detection of bound antibody was carried out using SuperSignal™ West Pico PLUS Chemiluminescent Substrate (Thermo Scientific) and X-ray film (Fujifilm), or by scanning with an Odyssey^®^ CLx (LI-COR Biosciences, Lincoln, NE), followed by immunoblot quantification using Image Studio Lite 5.2.5 (LI-COR).

### Microscopy

Preparation, fixation and labelling of cells for fluorescence and electron microscopy, acid phosphatase cytochemistry, preparation of thin sections for transmission EM (TEM) and immuno-EM of frozen sections, use of microscopes, image analysis and quantification were carried out as previously described (Bright et al., 2016), unless stated otherwise. For immunofluorescence microscopy, primary antibodies were used at 1:1000 dilution, except anti-VPS18 (1:50) and anti-YKT6 (1:500), with fluorescently conjugated secondary antibodies at 1:1000 dilution. For immuno-EM, primary antibodies were used at 1:100 dilution except for anti-VPS18 (1:50), with rabbit anti-mouse IgG (1:100) as a bridge for VPS35 labelling and PAG conjugates at 1:50 dilution. Confocal and live cell microscopy were carried out on Zeiss 780 and 880 confocal microscopes, and EM sections were examined with an FEI Tecnai G2 Spirit BioTwin TEM (Eindhoven, The Netherlands).

For high-pressure freezing and freeze substitution, an EMPACT2 high-pressure freezer (Leica, Milton Keynes, UK) was used to vitrify samples at −196°C and 2100 bar within 20 ms. Vitrified cell pellets were then freeze-substituted in a Leica AFS2 (Leica, Milton Keynes, UK) using 1% OsO_4_ and 0.1% uranyl acetate in acetone at −90°C for 5 h and the temperature was raised to 0°C at 5°C/h. The sample was then exchanged into Agar 100 resin (Agar Scientific, Stansted, UK) and polymerised at 60°C.

To flood the endocytic system, cells were incubated with 2 mg/ml HRP for 24 h. The cells were then washed with PBS and fixed with 2.5% glutaraldehyde/2% paraformaldehyde (PFA) in 0.1 M Na cacodylate buffer (pH 7.2) and the DAB reaction was performed by incubating with 1 mg/ml DAB in 0.1 M Tris-HCl buffer (pH 7.4) containing 0.3% H_2_O_2_ for 10 min at room temperature in the dark.

To investigate trafficking of newly synthesised LAMPs to MVBs, cells were transiently transfected with GFP-tagged rat LAMP1 (pEGFP-N1-lgp120, Addgene #62963) for 16 h using Lipofectamine. Cells were then fixed with 2% PFA in 0.1 M phosphate buffer (pH 7.4) and prepared for immuno-EM of frozen sections prepared by following the Tokuyasu technique, using mouse anti-LAMP1 (clone H4A3) followed by rabbit anti-mouse IgG or rabbit anti-GFP and PAG as previously described (Bright et al., 2016). Immunolabelling of VPS35 on frozen sections was performed using mouse anti-VPS35 (clone B-5, Santa Cruz Biotechnology).

For pre-embedding immunolabelling, cells were permeabilised by immersion into liquid nitrogen as described previously (Seaman, 2004). Cells were then fixed with 2% PFA in 0.1 M phosphate buffer (pH 7.4) washed with PBS and unreacted aldehydes were quenched with 50 mM NH_4_Cl. Pre-embedding immunolabelling was performed using rabbit anti-LAMP1 (ab24170) followed by PAG. The cells were then prepared for TEM using Agar 100 resin. For pre-embedding immunolabelling of Vps18 in CHMP6-depleted permeabilised cells, rabbit affinity-purified anti-Vps18 was detected using Fab fragments of goat anti-rabbit IgG conjugated to 1.4 nm nanogold particles and subsequently enhanced by using GoldEnhance™ EM plus, according to the manufacturer's instructions prior to processing for TEM.

BSA conjugated to 5 nm colloidal gold was prepared and endocytosed for 4 h followed by a 20-h chase as described previously (Bright et al., 1997). In some samples, for routine TEM, 1% tannic acid was used as a post-fixation mordant for subsequent lead citrate staining of the sections.

### Electron tomography

For electron tomography, tilt series acquisitions were performed on uranyl acetate and lead citrate-stained 250–300-nm thick resin-embedded sections. Data were obtained between −60° and +60° by using increments of one to two degrees and collected using Xplore3D (FEI, Eindhoven, The Netherlands) on a FEI Tecnai20 200 kV TEM or using Tomography 4.0 (FEI) on a Tecnai G2 Spirit BioTWIN TEM at 120 kV. Images were recorded using an Eagle 4 K CCD (FEI) or a Gatan^®^ US1000 2 K CCD. Tilt series alignments and back projection reconstructions were performed using eTomo (IMOD) software (University of Colorado, Boulder). Manual contours of features of interest within the tomography reconstructions were drawn using 3dmod (IMOD) software and images were exported to NIH Image J (v1.48r) for the generation of model animations.

## Supplementary Material

Supplementary information

Reviewer comments
