# Supplementary material for: Organelle tethering, pore formation and SNARE compensation in the late endocytic pathway
Source: J Cell Sci. 2021 May 27;134(10):jcs255463. doi: 10.1242/jcs.255463 (PMC8186482; doi:10.1242/jcs.255463)
Supplement: Supplementary information [file joces-134-255463-s1.pdf]

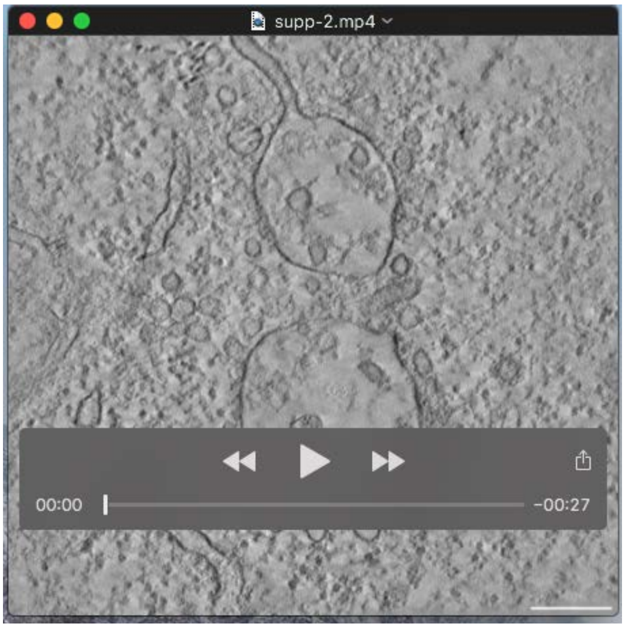

Movie 1: Electron tomography and 3D reconstruction of a thick section from a chemically-fixed VAMP7+VAMP8 double knockout HeLa cell revealing the accumulation of small vesicles (pink) surrounding two MVB's (cyan). Some of the vesicles have clearly-defined tethers (green). The flat clathrin patches (blue) are devoid of attached vesicles. Tubular elements apposed to, but not contiguous with, MVBs are likely to be smooth ER (white). Scale bar 200nm.

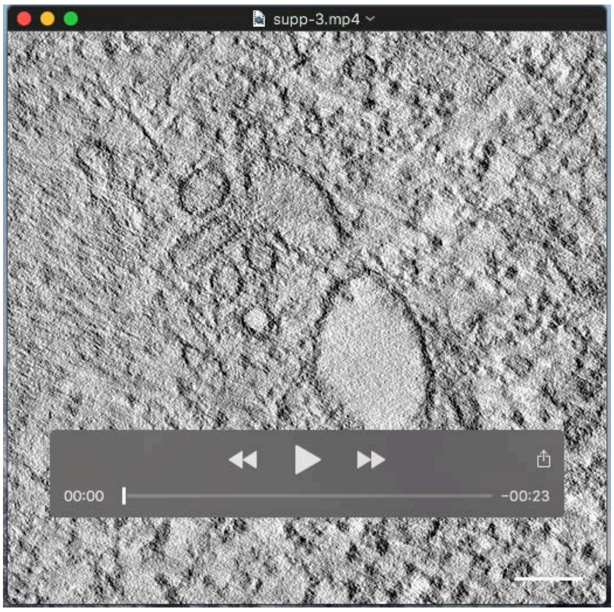

Movie 2: Electron tomography and 3D reconstruction of a thick section from a chemically-fixed VAMP7+VAMP8 double knockout HeLa cell revealing 'hourglass profiles' of MVB's (cyan) connected via small fusion pores and tethers (green). Scale bar 100nm.

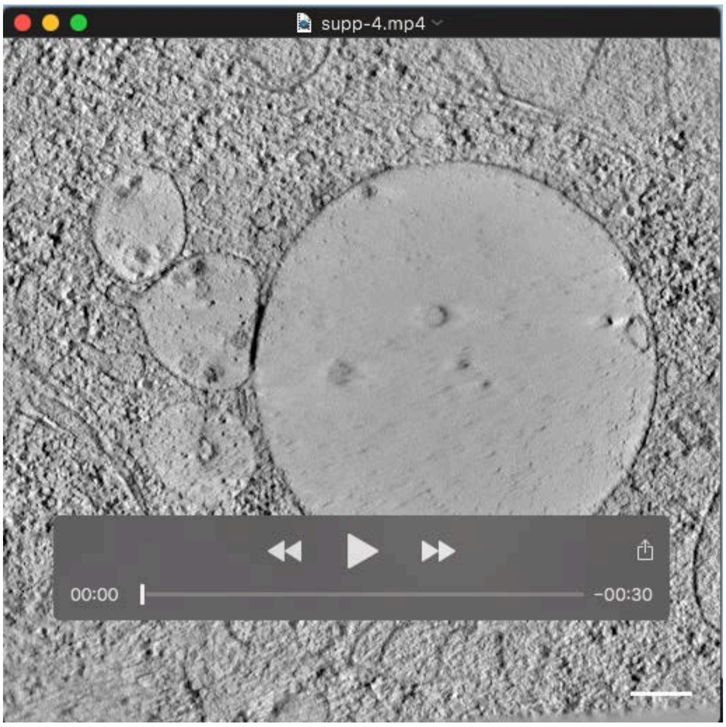

Movie 3: Electron tomography and 3D reconstruction of a thick section from a high pressure frozen and freeze-substituted HeLa cell in which CHMP6 has been depleted by treatment with siRNA. MVB's and the swollen endolysosome (cyan) are interconnected by numerous fusion pores at the periphery of the tethered regions (green). Scale bar 200nm.

Journal of Cell Science • Supplementary information

Supplementary Figures

Figure S1

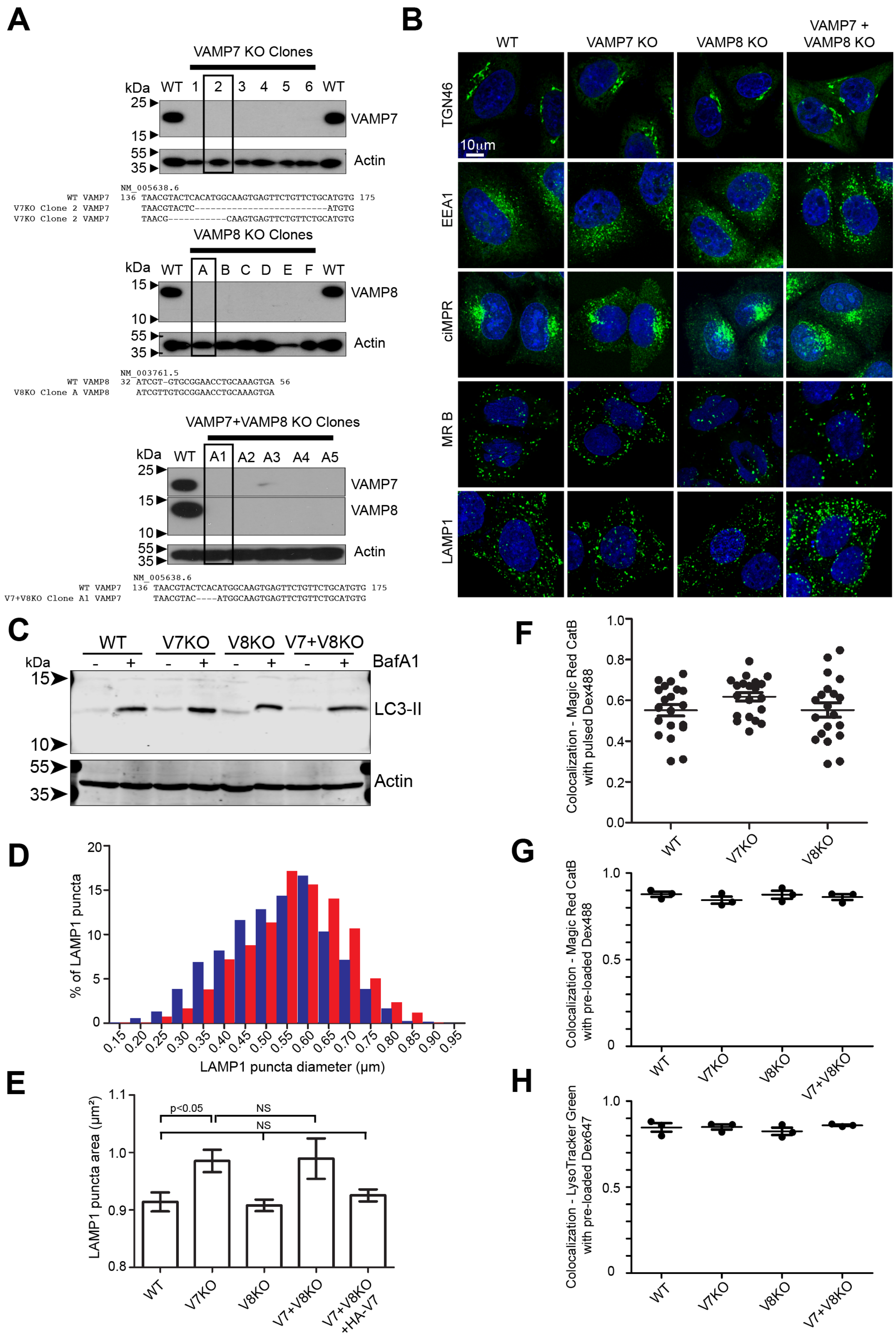

Journal of Cell Science • Supplementary information

Figure S1. Characteristics of HeLa cells following knockout of VAMP7 and VAMP8. (A) Immunoblots showing loss of VAMP7 and VAMP8 in single and double knockout cell lines. For all further experiments the lines selected were VAMP7 KO clone 2, VAMP8 KO clone A and the double knockout VAMP7+VAMP8 KO clone A1(VAMP7 knocked out in VAMP8 KO clone A). DNA sequences showing deletions/insertions are shown based on sequences NM_005638.6 for VAMP7 and NM_ 003761.5 for VAMP8 with bases numbering from the start of the open reading frame. **(B)** Confocal fluorescence microscopy images of wild type and knockout HeLa cells showing immunofluorescence localization of marker proteins (green) in paraformaldehyde-fixed cells (or live cells for hydrolysed Cathepsin B Magic Red® substrate, MR B). Nuclei were labelled with Hoechst 33342(blue). **(C)** Representative immunoblots of LC3-II in wild type HeLa cells and VAMP7+VAMP8 KO clone A1. Bafilomycin A (BafA1, 400nM) for 8h was a control for autophagosome accumulation. **(D)** Representative experiment showing rescue of LAMP1-positive puncta diameter by over-expression of HA-tagged VAMP7 (blue) in VAMP7+VAMP8 KO clone A1 (red). **(E)** Rescue of LAMP-positive organelle size by over-expression of HA-tagged VAMP7 in VAMP7+VAMP8 KO clone A1. Data shown are means±SEM of LAMP1-positive puncta areas from three independent experiments with 10 LAMP1-stained cells analysed in each cell type/condition as in Fig. 1B and statistical analysis by two-tailed unpaired t-test. **(F)** Colocalization (Manders' coefficient) of endocytosed fluorescent dextran (loaded for 2 h, chased for 1h) with hydrolysed Cathepsin B Magic Red® substrate using images captured by live-cell confocal microscopy in wild type and VAMP7 or VAMP8 single knockout HeLa cells. Representative experiment shown with 20 cells total per condition. **(G)** Colocalization (Manders' coefficient) of hydrolysed Cathepsin B Magic Red® substrate with preloaded fluorescent dextran (loaded for 4 h, chased for 20h) in wild type and VAMP7 and VAMP8 single or double knockout HeLa cells. Means±SEM of 3 independent experiments with five fields analysed in each experiment, ≥ 30 cells total per condition. **(H)** Colocalization (Manders' coefficient) of lysotracker with preloaded fluorescent dextran (loaded for 4 h, chased for 20h) in wild type and VAMP7 and VAMP8 single or double knockout HeLa cells. Means±SEM of 3 independent experiments with five fields analysed in each experiment, ≥ 30 cells total per condition.

Journal of Cell Science • Supplementary information

Figure S2

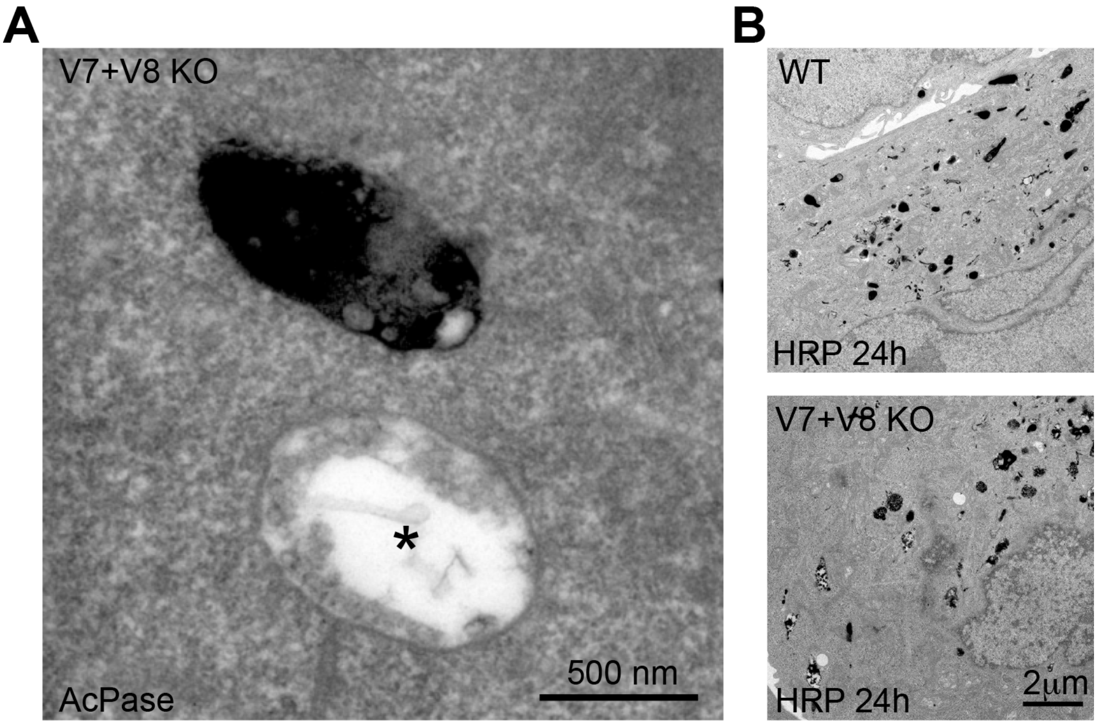

Figure S2. Ultrastructural features of HeLa cells following double knockout of VAMP7 and VAMP8. (A) A representative image of acid phosphatase reactivity within late endocytic organelles of an unstained VAMP7+VAMP8 double knockout HeLa cell. Note that both the acid phosphatase-positive MVB (endolysosome) and the acid phosphatase-negative MVB (*, late endosome) are devoid of acid phosphatase reactivity in any surrounding small vesicles. **(B)** Representative low power thin EM sections of WT and VAMP7+VAMP8 double knockout HeLa cells following continuous uptake of HRP for 24h to label all endosomal/lysosomal compartments. 30 low power images per sample were analysed by systematic rastering and scored using a 2 μm grid to count intersections and calculate the volume fraction occupied by HRP-positive organelles, which was 1.1% in the WT cells and 0.8% in the VAMP7+VAMP8 double knockout cells. The number of HRP-positive organelle profiles in WT cell sections was 0.19 per μm^2 and in VAMP7+VAMP8 double knockout cells, 0.12 per μm^2. The change in number of the HRP-positive compartments was consistent with the reduction in number of LAMP1-positive puncta observed by immunofluorescence confocal microscopy (Fig. 1A).

Journal of Cell Science • Supplementary information

Figure S3

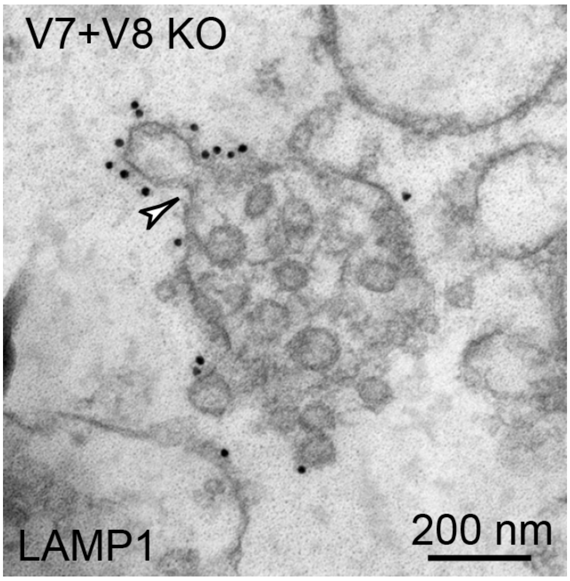

Figure S3. LAMP1 labelling of an omega-shaped profile. Example of an MVB from a VAMP7+VAMP8 double knockout cell and pre-embedding labelling of endogenous LAMP1, showing labelling of tethered small vesicles and, in addition, labelling of the limiting membrane of an omega-shaped profile connected to the MVB via a fusion pore (white arrowhead).

Journal of Cell Science • Supplementary information

Figure S4

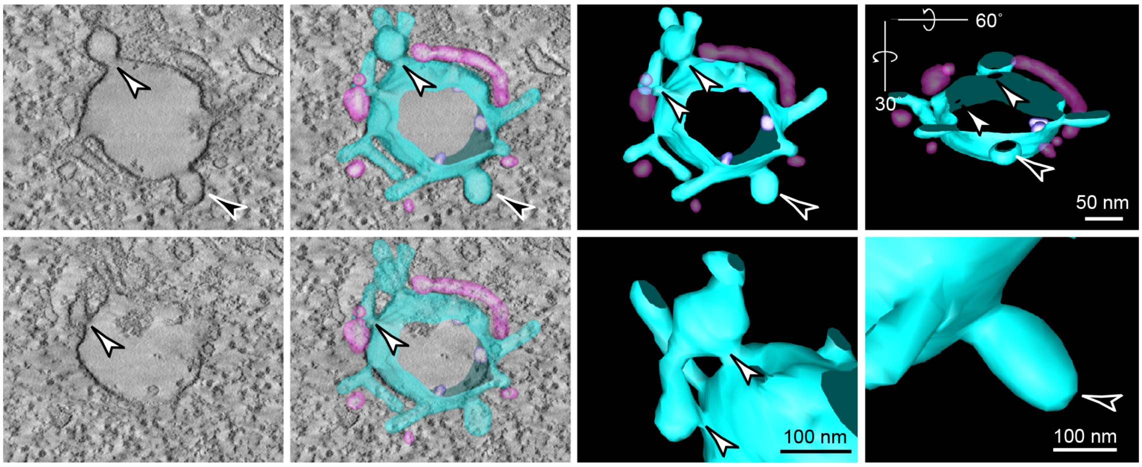

Figure S4. Morphological features of HeLa cells following double knockout of VAMP7 and VAMP8. Single tomographic slices from a reconstruction of a thick section of a chemically-fixed VAMP7+VAMP8 double knockout cell revealing two fusion pores (white arrowheads) between an MVB and a connected tubular endosome. In addition, a fusion pore between the MVB and an omega shaped very large vesicle/organelle (black arrowhead) is also captured. The fusion pores were clearly revealed by rotating and projecting a slice of the model to show more of the morphology of the connected organelles.

Figure S5

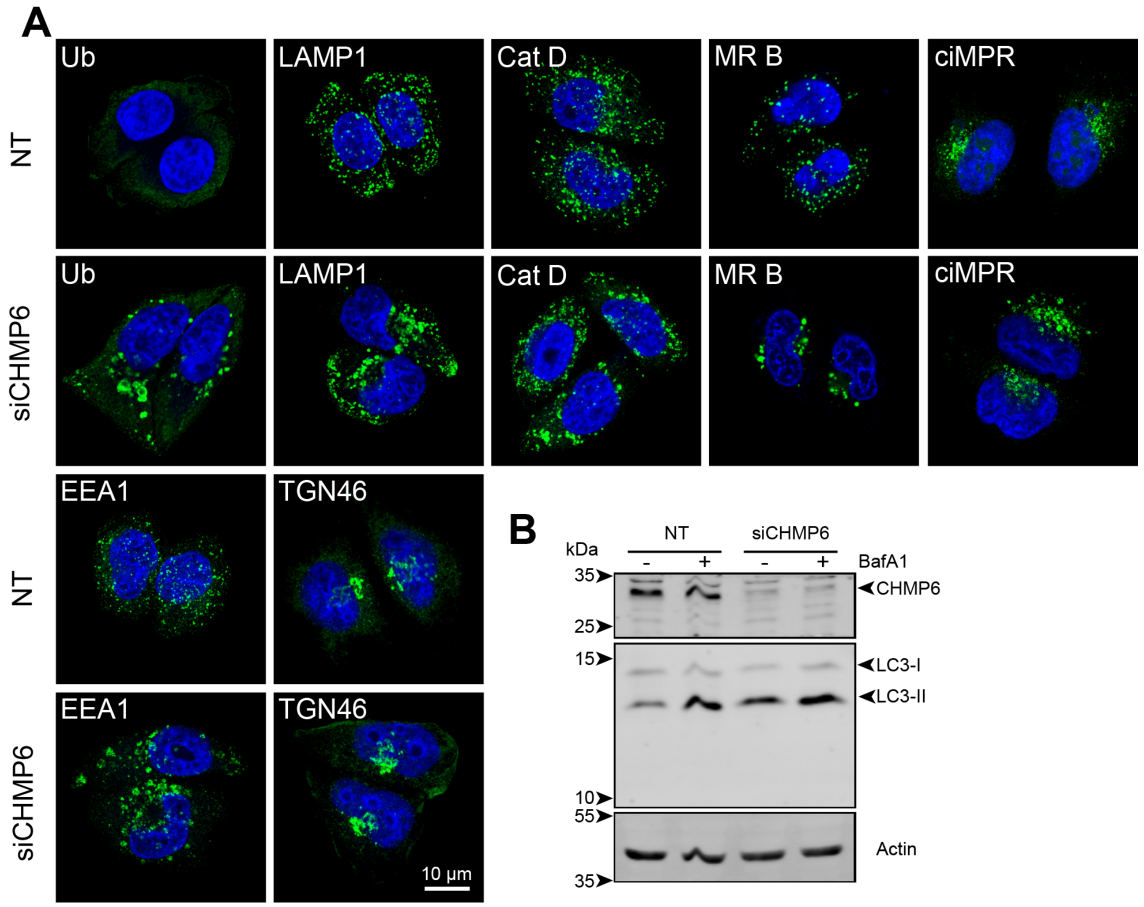

Figure S5. Characteristics of HeLa cells following depletion of CHMP6. **(A)** Confocal fluorescence microscopy images of control and CHMP6-depleted HeLa cells showing immunofluorescence localization of marker proteins (green) in paraformaldehyde-fixed cells or hydrolysed Cathepsin B Magic Red® substrate (MR B) in live cells. Nuclei were labelled with Hoechst 33342 (blue). Ub, ubiquitin; Cat D, Cathepsin D. (**B**) Representative immunoblots of LC3-II in wild type cells and cells depleted of CHMP6. Bafilomycin A (BafA1, 400nM) for 8h was a control for autophagosome accumulation. The observed increase in LC3-II concentration (and P62, data not shown) following CHMP6 depletion is consistent with the increased ubiquitination of the late endocytic organelles (Fig. S5A and immunoEM data not shown) and indicates disruption of the autophagic pathway.

Journal of Cell Science • Supplementary information

Figure S6

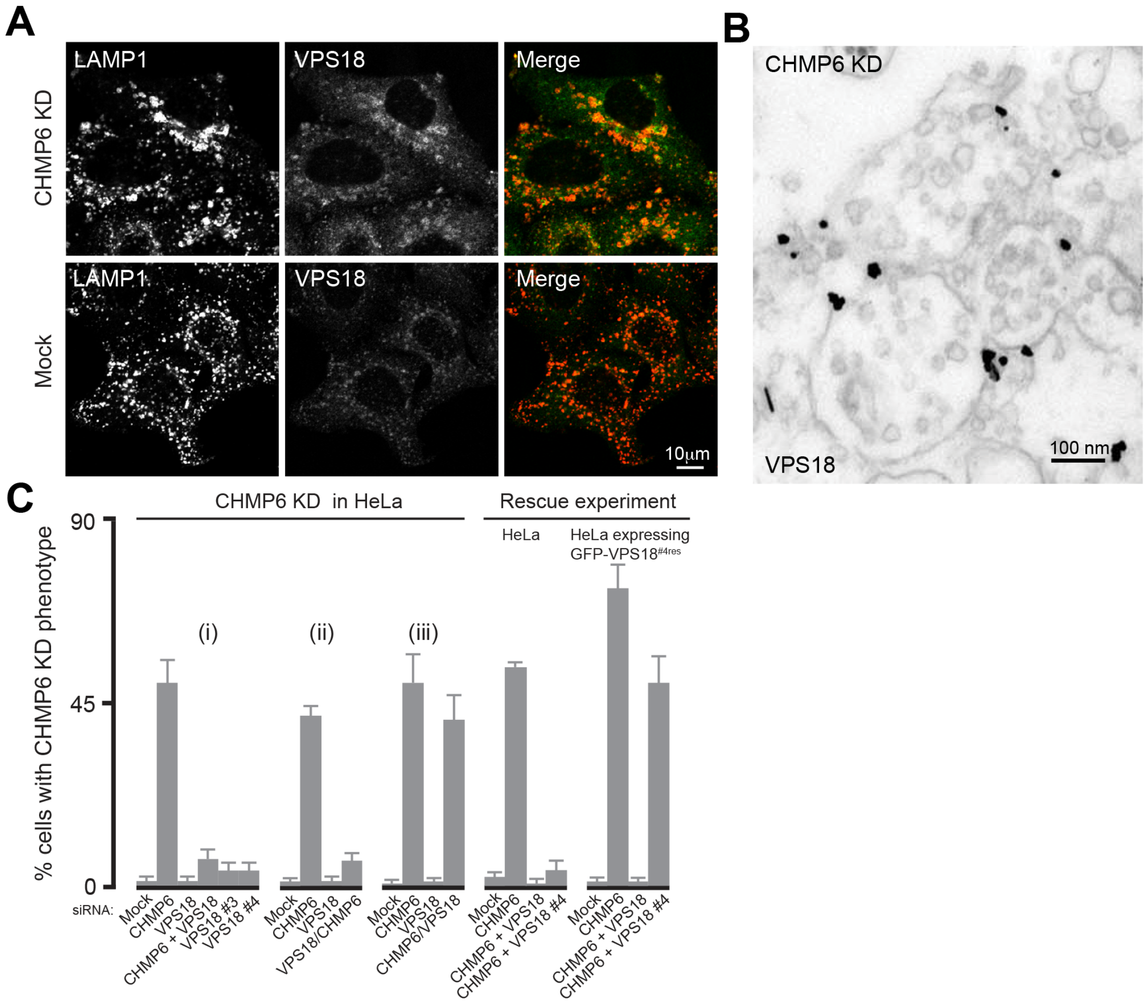

Figure S6. Characteristics of CHMP6-depleted HeLa cells and of filamentous tethers and fusion pores in these cells. (A) Confocal immunofluorescence localization of endogenous VPS18 and LAMP1 in paraformaldehyde-fixed CHMP6-depleted (CHMP6 KD) and non-targeting siRNA-treated (Mock) HeLa cells. Images from CHMP6 KD and Mock cells were collected with identical detector settings. In Merge, LAMP1, red and VPS18, green. (**B**) ImmunoEM localization of endogenous VPS18 at clustered MVBs in CHMP6-depleted HeLa cells following pre-embedding labeling and gold enhancement. (**C**) Data showing the effect, on the appearance the swollen ubiquitinated organelle phenotype in HeLa cells, of depletion of VPS18 at the same time (i), before (ii) or after (iii) the depletion (KD) of CHMP6. siRNA pools were used except when individual VPS18 siRNA duplexes #3 and #4 were used. In the rescue experiment HeLa cells or HeLa cells stably expressing GFP-tagged VPS18 that was resistant to depletion by VPS18 siRNA duplex #3 were used. Data shown as mean ±SEM (≥ 50 cells).

Journal of Cell Science • Supplementary information

Figure S7

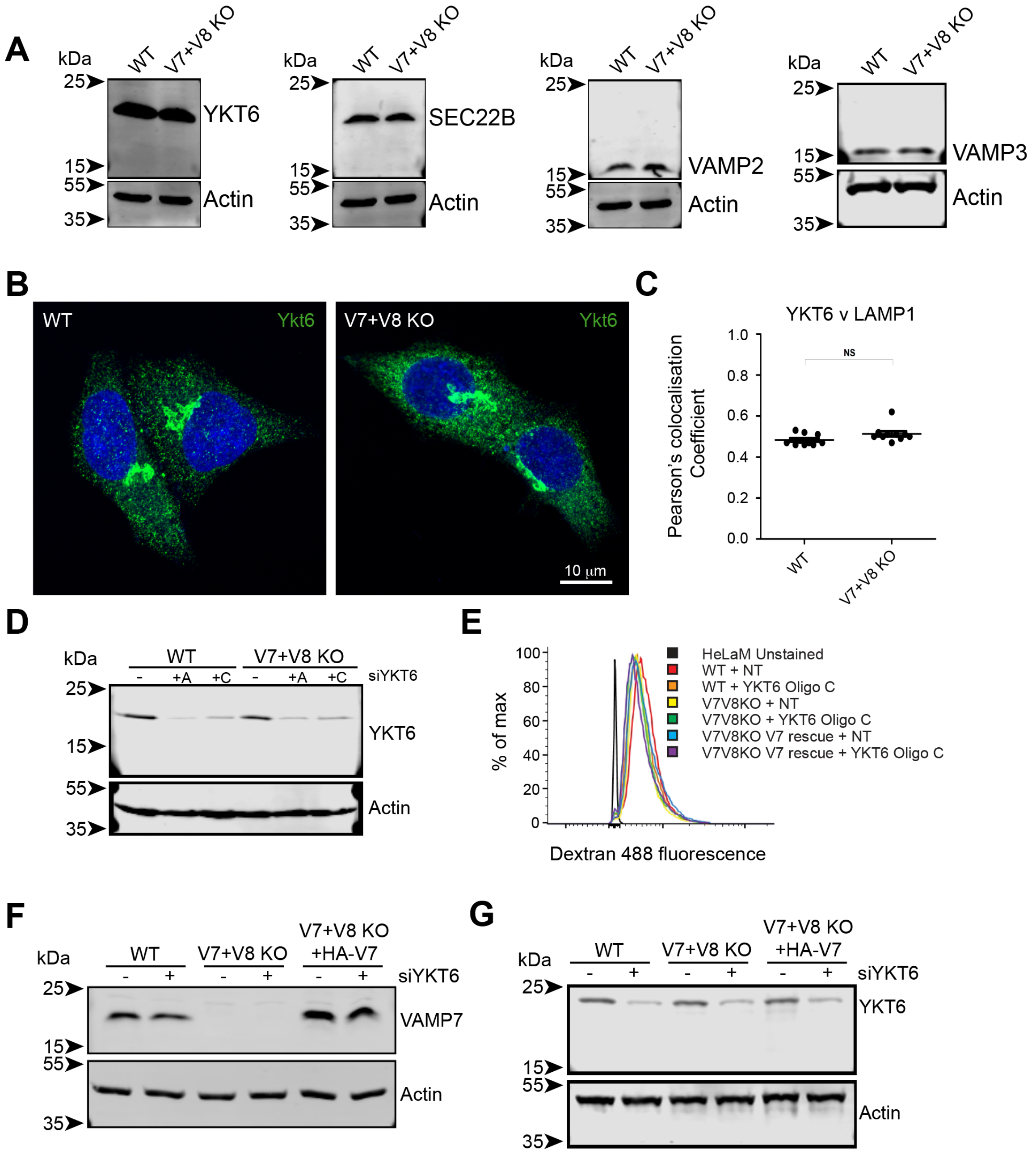

Figure S7. The effects of YKT6 depletion on VAMP7+VAMP8 double knockout cells. (A) Representative immunoblots of R-SNAREs in wild type (WT) and VAMP7+VAMP8 double knockout HeLa cells. **(B)** Confocal fluorescence microscopy images of endogenous YKT6 (green) distribution in wild type (WT) and VAMP7+VAMP8 double knockout cells. Nuclei were labelled with Hoechst 33342 (blue). **(C)** Quantification of colocalization of YKT6 with LAMP1 in 10 wild type (WT) and 10 VAMP7+VAMP8 double knockout (V7+V8 KO) cells. The mean Pearson's colocalization coefficients are shown and there was no statistically significant difference (NS) between the two cell types when analysed by two-tailed

Journal of Cell Science • Supplementary information

unpaired t-test. **(D)** Representative immunoblots showing depletion of YKT6 in wild type (WT) and VAMP7+VAMP8 double knockout cells, after the single transfection protocol with YKT6 siRNA oligoC. **(E)** Representative FACS analysis of dextran Alexa Fluor 488 fluorescence after uptake by wild type (WT) and VAMP7+VAMP8 double knockout cells ±YKT6 depletion with YKT6 siRNA oligoC and ± 'rescue' with HA-tagged VAMP7. 30,000 cells were measured per condition. Data were analysed using FLOWJO software and histogram overlays are displayed as % of max, scaling each curve to mode = 100%. NT, non-targeting control oligonucleotide. **(F)** Representative immunoblots of VAMP7 in wildtype HeLa cells and in VAMP7+VAMP8 double knockout HeLa cells and ± 'rescue' with HA-tagged VAMP7, from experiments shown in Fig. 7B. **(G)** Representative immunoblots of YKT6 in wildtype HeLa cells and in VAMP7+VAMP8 double knockout HeLa cells and ± 'rescue' with HA-tagged VAMP7, from experiments shown in Fig. 7B.

Journal of Cell Science • Supplementary information
