# Supplementary material for: Organelle tethering, pore formation and SNARE compensation in the late endocytic pathway
Source: J Cell Sci. 2021 May 27;134(10):jcs255463. doi: 10.1242/jcs.255463 (PMC8186482; doi:10.1242/jcs.255463)
Supplement: Reviewer comments [file joces-134-255463_review_history.pdf]

Journal of Cell Science

Organelle tethering, pore formation and SNARE compensation in the late endocytic pathway

Luther J. Davis, Nicholas A. Bright, James R. Edgar, Michael D. J. Parkinson, Lena Wartosch, Judith Mantell, Andrew A. Peden and J. Paul Luzio
DOI: 10.1242/jcs.255463

Editor: Jennifer Lippincott-Schwartz

Review timeline

Original submission:	12 October 2020
Editorial decision:	29 December 2020
First revision received:	24 March 2021
Accepted:	14 April 2021

Original submission

First decision letter

MS ID#: JOCES/2020/255463

MS TITLE: Organelle tethering, pore formation and SNARE compensation in the late endocytic pathway

AUTHORS: Luther J Davis, Nicholas A Bright, James R Edgar, Michael DJ Parkinson, Lena Wartosch, Judith Mantell, Andrew A Peden, and J. Paul Luzio
ARTICLE TYPE: Research Article

We have now reached a decision on the above manuscript.

To see the reviewers' reports and a copy of this decision letter, please go to: https://submit-jcs.biologists.org and click on the 'Manuscripts with Decisions' queue in the Author Area. (Corresponding author only has access to reviews.)

As you will see, the reviewers gave favourable reports but raised some critical points that will require amendments to your manuscript. I hope that you will be able to carry these out, because I would like to be able to accept your paper. Please address all of the concerns of the reviewers. I don't think it is necessary to split your study into two separate papers, as recommended by reviewer #2. Rather, by melding some of the supplementary data into the main figures, as suggested by reviewer #1, you should be able to clarify the two story lines.

We are aware that you may be experiencing disruption to the normal running of your lab that makes experimental revisions challenging. If it would be helpful, we encourage you to contact us to discuss your revision in greater detail. Please send us a point-by-point response indicating where you are able to address concerns raised (either experimentally or by changes to the text) and where you will not be able to do so within the normal timeframe of a revision. We will then provide further guidance. Please also note that we are happy to extend revision timeframes as necessary.

Please ensure that you clearly highlight all changes made in the revised manuscript. Please avoid using 'Tracked changes' in Word files as these are lost in PDF conversion.

© 2021. Published by The Company of Biologists under the terms of the Creative Commons Attribution License (https://creativecommons.org/licenses/by/4.0/).

I should be grateful if you would also provide a point-by-point response detailing how you have dealt with the points raised by the reviewers in the 'Response to Reviewers' box. Please attend to all of the reviewers' comments. If you do not agree with any of their criticisms or suggestions please explain clearly why this is so.

Reviewer 1

Advance summary and potential significance to field

In this manuscript Davis, Bright and collaborators investigate in detail mechanisms involved in pore formation between late endocytic organelles essential for delivery of contents to lysosomes.

The data presented is of very high quality with superb electron microscopy analysis following the respective silencing of VAMP7/VAMP8. If small vesicles accumulate in close apposition to the late endocytic organelles such depletion that finally doesn't impair the delivery because of compensation probably by YKT6.
Of interest are the filamentous tethers that appear to be required "pre fusion"that could be composed of tethering factors such as HOPS complexes.
The authors also investigate the consequences of depleting the ESCRT III subunit CHMP6 that was shown previously by the authors to induce clustering of late endocytic organelles.
Summary it is the first analysis at high resolution of pore forming zones between organelles

Comments for the author

In the opinion of this reviewer the paper is of interest as proposing a conceptual model for the mechanisms underlying kiss and run between organelles, an essential for proper organelle maturation

I have nevertheless some points that could be clarified in the present manuscript as a lot of interesting data is depicted in the supplementary information. I am wondering whether some of the data would not been important to include somewhere in the main manuscripts

- As compared to control WT cells Upon KO of VAMP7/VAMP8 the Lamp1 positive organelles appear more abundant?(supp information) Impaired fusion or impaired retrieval? as discussed below (increased number of emerging tubules?)
- In the main manuscript the authors do not mention the tubules emerging from endosomes in VAMP7/VAMP8 KO cells. Are those more abundant in the KO cells?
- If so would this be interesting to discuss. They are clearly observed by electron tomography. The authors show nicely a labeling for the retromer subunit VPS35 (see comment above)
- KO of VAMP7/VAMP8 does not impair the delivery of hydrolases though It is not clear for me whether a cargo as for example EGFR could be delivered or whether its signaling is not stopped upon such KO?
- The authors show nicely the filamentous tethers. In one experiment the authors silence VPS33A without really describing or commenting on the reason of such experiment and knowing that the HOPS complex is composed of more subunits.

-I am also wondering until what point the increased ubiquitination is directly linked then to the accumulation of the tethers at the endosomal membrane (as observed with CHMP6 depletion). Is there any hint ? an aside question is why CHPM6 and not another ESCRT III subunit?

In all I think it is an interesting descriptive study nicely documented with high resolution data that supports a "take home message" that the authors do not over interpret.
In brief the paper would benefit from some of the data presented in the supp data that is pretty interesting. Some experiments are not really described and theb reader doesn t fully understand the rational (as stated above).

© 2021. Published by The Company of Biologists under the terms of the Creative Commons Attribution License (https://creativecommons.org/licenses/by/4.0/).

Reviewer 2

Advance summary and potential significance to field

In this paper the authors investigate the relationship between tethering and fusion pore formation. By knocking out SNARE proteins VAMP7, VAMP8 and ESCRT protein CHMP6 they block the fusion process and induce increased numbers of tethering profiles. By a combination of EM techniques they investigate how these tethers are positioned relative to fusion pores.

The problem of this paper is that it contains a mixed message. On the one hand there is a description of defects induced by VAMP7/VAMP8 KO: no interference with late endosome – lysosome fusion but instead accumulation of LAMP carriers.
These interesting observations raise questions on the current view on the role of VAMP7 and VAMP8 in lysosome fusion. However, they do not contribute to the main question of the paper; how tethers and fusions processes are related. Also the induction of tether profiles, which is why they did these experiments, is only low when compared to CHMP6 KO cells. KO of CHMP6 does give an endocytosis defect and results in many endosomal tethering profiles. However, though the EM is of high quality and gives a first insight into where pores are formed relative to filament tethering, the morphological information is not translated into mechanism and the paper does not add novel information to our understanding of the fusion process.

Overall, these are two stories at a preliminary state. My suggestion would be to split the paper and extend experiments in the direction of 1. Characterisation of the VAMP7/VAMP8 KO phenotype and 2. Using CHMP6 to investigate in more detail the tethering – pore formation.

Comments for the author

General comment
It took a while before I understood what authors meant with ‘pores’ and how to recognise these in the pictures. It should be clearly defined how they identify a membrane indent as fusion pore. In case of the hourglass profiles the authors event state that they are ‘likely in the process of fusion’. Apart from defining fusion, how do they distinguish between fusion and fission of membranes?

VAMP7/VAMP8 KO phenotype
In VAMP7/VAMP8 KO cells the authors describe hourglass profiles associated with what they identify as ‘late endosomes’ (Figure 3A). However, these are very empty organelles and in my opinion early endosomes. What are the author’s criteria for naming organelles early or late endosomes? VAMP7 and VAMP8 are late endosome -lysosomal SNAREs. Why would KO of VAMP7 and VAMP8 block fusion with early endosomes? In figure 3A. it takes quite some goodwill to see the tethers in the EM pictures that are represented in green in the model. The organelles in Fig. 3C,D (I would say autolysosomes) are definitely a later stage of organelle maturation than the organelles in Figure 3A and B, and they show a different type of tethering (arrays). All in all these data raise too many questions on what the images exactly represent.

How do the authors explain the (mildy) altered fluorescent LAMP staining pattern in these cells when there is no obvious effect on endolysosomal morphology?

Authors claim that there is no alteration of autophagy and no changes in LC3 in VAMP7/VAMP8 KO cells. Both VAMP7 and VAMP8 have been identified several times as players in the autophagy process (fusion of lysosomes-autophagosomes).
Therefore, they should show data to confirm this statement, instead of “data not shown”. The finding is intriguing since it again questions the role of VAMP7/VAMP8 in late endosome – lysosome fusion.

It is interesting that depletion of YKT6 impairs the delivery of Dextran to enzymatically active organelles in VAMP7-VAMP8 KO cells. However, the lack of a proper rescue assay is a gap. Rescue with VAMP7 is not appropriate since this rescues VAMP7 KO and not YKT6 depletion. Figure 5A and 5B —why are there only 3 data points? All cells should be represented, not only the averages within an experiment. In general, I don’t think this part is thoroughly done, maybe depletion of YKT6

© 2021. Published by The Company of Biologists under the terms of the Creative Commons Attribution License (https://creativecommons.org/licenses/by/4.0/).

blocks the endocytosis delivery to lysosomes at an earlier/ different stage in the endocytic pathway?

CHMP6 KO cells CHMP6 KO cells In the CHMP6 KO cells authors see MVBs tethering to enlarged endo-lysosomes. What is the nature of these MVBs? Since these cells lack the ESCRT complexes involved in the making of intraluminal vesicles.
In CHMP6 KO cells there are many tethering profiles judged by the presence of electrondense staining between compartments. A weakness in the paper is the identification of these electrondense structures. This is done on basis of morphology, the criteria for which are not made clear by the authors and the interpretation of which leaves room for dispute. Authors tried but failed to label Vps18 with pre-embedding EM. I suggest to try immunoEM of cryosections, as they did for Vps35 labeling.

Depletion of CHMP6 causes extensive tethering of MVBs to each other and to swollen endolysosomes. Fig. 4B,C and Movie 3 are interesting images, giving a first insight into where pores are formed relative to filament tethering. A next step could by cryo-EM

In CHMP6-depleted HeLa cells, the human HOPS protein VPS18 associates with swollen LAMP-1 endolysosomes. Depletion of VPS18 by IF prevents formation of these swollen structures. However, the authors do not show if this also prevents the formation of tethers between MVBs and endolysosomes, which would be a key experiment for this line of investigation.

Pores are preferentially formed near the edge of tethered regions. Pore expansion is coupled to the release of the filamentous tethers. However, authors cannot show what these tethers are. That would be a strong result.

Reviewer 3

Advance summary and potential significance to field

In this paper the authors show, surprisingly, that double knockout of two SNAREs, VAMPs 7 and 8, that have previously been implicated in endosome:lysosome fusion, induces a comparatively mild cellular phenotype and does not affect delivery of endocytic cargo to acid hydrolase positive lysosomes. They show that this is because another SNARE, YKT6, compensates for loss of VAMPS 7 and 8. The VAMP7/8 knockout cells do however show morphological abnormalities, specifically an accumulation of vesicles carrying newly synthesised LAMP1 tethered to endosomes and the presence of hourglass profiles where adjacent endosomes and lysosomes are attached by filamentous structures and connected by pores. The authors further investigate the relationship between the pores and tethers by depleting cells of CHMP6, previously shown to accumulate tethered endosomes and lysosomes, and show that pore formation occurs at the edge of tethered regions and suggest that full fusion requires expansion of the pore. The delivery of cargo to lysosomes for degradation is a fundamental process crucial for cellular homeostasis and yet there is still much that we don’t know about this process. I believe this paper would be of wide interest not only to lysosome biologists but also those interested in organelle interaction and membrane fusion in general, since tethering, followed by SNARE-dependent fusion, is a universal mechanism for membrane transport.

Comments for the author

The paper is well written, the data is convincing and the electron microscopy and tomography is spectacular.
The following minor points should however be addressed:

An assay measuring delivery of endocytosed dextran to lysosomes that stain positive for a magic red cathepsin B substrate is used. This assay relies on cathepsin B being delivered to lysosomes, even though this occurs via the endocytic pathway so anything that inhibits delivery of dextran is likely to inhibit delivery of newly synthesised cathepsins too. Given that the MR signal requires the cathepsins to be active then a positive MR signal represents a lysosome that has received cathepsin so clearly there are lysosomes present in VAMP7/8 KO cells that can receive both dextrans and

© 2021. Published by The Company of Biologists under the terms of the Creative Commons Attribution License (https://creativecommons.org/licenses/by/4.0/).

cathepsins. In VAMP7/8 KO cells depleted of YKT6 there is an inhibition of delivery of dextrans to MR positive lysosomes so I don't quite understand how the cathepsin is being delivered if the dextrans are not. Is the turnover of cathepsins sufficiently slow that there is sufficient cathepsin synthesised before knockdown of YKT6 to cleave the MR substrate? I think this point should be discussed/clarified in the text.

Figure 2B: Clathrin patches are indicated on the tomogram in dark blue but are quite hard to see in the images- maybe it would be more accurate to describe them as 'resembling the clathrin coats seen on flattened regions of the endosomal limiting membrane' Also, are the authors sure about that the tube like structure on the right of the vacuole in Figure 2B (running parallel to the limiting membrane) should be coloured pink? On the movie it looks like it could be ER to me (especially as it is sitting by a tubular extension of the limiting membrane and ER:endosome contacts are believed to mark sites of recycling tubule scission).

Figure 4A: A lower magnification view would be helpful to see the enlarged endolysosome.

First revision

Author response to reviewers' comments

Editor: *As you will see, the reviewers gave favourable reports but raised some critical points that will require amendments to your manuscript. I hope that you will be able to carry these out, because I would like to be able to accept your paper. Please address all of the concerns of the reviewers. I don't think it is necessary to split your study into two separate papers, as recommended by reviewer #2. Rather, by melding some of the supplementary data into the main figures, as suggested by reviewer #1, you should be able to clarify the two story lines.*

We are very grateful for the favourable reports and for the suggestion that we meld some of the supplementary data into the main figures in order to clarify our manuscript. Whilst we have kept the content of previous figures 1 and 5 as they were (now as new figures 1 and 7), we have expanded what were figures 2-4 to become new figures 2-6 and included many EM panels from the supplementary figures in the Supplementary Information that accompanied our original submission. After our point-by-point responses to the reviewers below, we have summarised the changes to the manuscript and include in that summary two tables that show the panels that have been moved (or added) to the new figures (and removed from the new set of supplementary figures). The places where we have made significant alterations to the wording of the text are highlighted in grey in the revised manuscript.

Reviewer 1 Comments for the Author:
In the opinion of this reviewer the paper is of interest as proposing a conceptual model for the mechanisms underlying kiss and run between organelles, an essential for proper organelle maturation. I have nevertheless some points that could be clarified in the present manuscript as a lot of interesting data is depicted in the supplementary information. I am wondering whether some of the data would not been important to include somewhere in the main manuscript.

We have now moved a significant amount of data from the supplementary information into the main figures. The moves are detailed in the tables below and we refer to the new figure numbers/legends in our responses.
-As compared to control WT cells Upon KO of VAMP7/VAMP8 the Lamp1 positive organelles appear more abundant?(supp information) Impaired fusion or impaired retrieval? as discussed below (increased number of emerging tubules?).

We have no evidence for a significant change in abundance of LAMP1+ve organelles in the VAMP7+VAMP8 double knockout (V7+V8KO). Our initial immunofluorescence microscopy analysis of the V7+V8KO cells indicated what we have described as a subtle alteration in the distribution of the late endosomal/lysosomal marker LAMP1. It was only when we measured the diameters of

© 2021. Published by The Company of Biologists under the terms of the Creative Commons Attribution License (https://creativecommons.org/licenses/by/4.0/).

LAMP1+ve puncta, that we were able to show a significant change in the V7+V8KO cells. Initially we had thought there might be a reduced abundance of LAMP1+ve organelle compared to those in wild type cells, as seen for example in the cells shown in Fig 1A, but there were clearly cells in which this was not the case as for example one of the cells shown in the bottom right panel of Fig. S1B, which, as the reviewer indicates, shows increased abundance compared to the WT cell in the left-hand panel. We apologise for the confusion caused by mentioning 'what appeared to be fewer labelled puncta per cell' at the start of our Results section and we have removed these words in the revised manuscript (Page 4). In the legend to Fig 1B, we provide the mean diameters of the LAMP1+ve puncta in each KO cell line, to show the significantly increased mean diameters in the V7KO and V7+V8KO cells. These numbers are reinforced by the data in supplementary Fig. S1E, which show the mean areas of LAMP1+ve puncta in each KO cell line.
-In the main manuscript the authors do not mention the tubules emerging from endosomes in VAMP7/VAMP8 KO cells. Are those more abundant in the KO cells?
-If so would this be interesting to discuss. They are clearly observed by electron tomography. The authors show nicely a labeling for the retromer subunit VPS35 (see comment above)

We apologise for not saying more about the tubules emerging from endosomes in V7+V8KO cells in the original manuscript. On Page 7 of the revised manuscript we now explain that there was no obvious change in the frequency or disposition of the VPS35+ve tubules in the V7+V8KO cells and explain their likely role in recycling membrane proteins to the TGN or plasma membrane. We have moved the EM images showing the VPS35 (retromer subunit) labeling of these tubules from supplementary information into the new revised main Fig 3D.
-KO of VAMP7/VAMP8 does not impair the delivery of hydrolases though It is not clear for me whether a cargo as for example EGFR could be delivered or whether its signaling is not stopped upon such KO?

Although it is clear that hydrolases are present in the endolysosomes of the V7+V8KO cells and that endocytic delivery of fluorescent-labelled dextran to these organelles is not prevented, we have not studied delivery of other cargoes including EGFR, nor investigated EGFR signaling in these cells. The focus of our present manuscript is on organelle tethering and pore formation. We do plan to study the V7+V8KO cells further in the future including a more detailed analysis of cargo delivery to endolysosomes, However, over the past 12 months our ability to do experiments has been seriously impeded by periods of lab closure and reduced occupancy as a result of government restrictions in response to the covid pandemic.
-The authors show nicely the filamentous tethers. In one experiment the authors silence VPS33A without really describing or commenting on the reason of such experiment and knowing that the HOPS complex is composed of more subunits.

We thank the reviewer for this comment and have amended our manuscript to explain our use of silencing VPS33A as a control in our experiments investigating endocytic delivery of fluorescent-labelled dextran to hydrolase+ve endolysosomes. In our revised manuscript we have included on Page 5 the sentence 'in these experiments, the knockdown of VPS33A was used as a positive control (Fig. 1C), because we have previously shown that depletion of this or other proteins of the HOPS complex, blocks endocytic delivery to the Magic Red®- positive organelles (Wartosch et al., 2015).'
-I am also wondering until what point the increased ubiquitination is directly linked then to the accumulation of the tethers at the endosomal membrane (as observed with CHMP6 depletion). Is there any hint ? an aside question is why CHPM6 and not another ESCRT III subunit?

At present we do not have any further data that throws light on these important questions. As we explain in our revised manuscript (Page 8), our focus on CHMP6 knockdown was because after this treatment we had observed the development of a swollen endolysosomal phenotype, often with associated clusters of MVB when we were previously studying the involvement of ESCRT proteins in down-regulation of virally ubiquitinated MHC Class I (Parkinson et al., 2015). Interestingly, it was only CHMP6 and not any other ESCRT-III protein that was required for the down-regulation of MHC Class I. Although we have not undertaken a detailed study of any morphological effects of depleting other ESCRT-III proteins, we are aware of reports in the literature suggesting a regulatory role for CHMP2B, CHMP3 and CHMP5 in endosome-lysosome fusion, distinct from their role in MVB formation and we cited these in the Discussion of our 2015 paper.
In all I think it is an interesting descriptive study nicely documented with high resolution data that supports a "take home message" that the authors do not over interpret.

© 2021. Published by The Company of Biologists under the terms of the Creative Commons Attribution License (https://creativecommons.org/licenses/by/4.0/).

In brief the paper would benefit from some of the data presented in the supp data that is pretty interesting. Some experiments are not really described and theb reader doesn t fully understand the rational (as stated above).

As explained above, we have moved a significant amount of data from the supplementary information into the main figures in the revised manuscript and we hope that this, together with the changes to the text, has made the rationale of our study clearer.

Reviewer 2 Comments for the Author:
General comment
It took a while before I understood what authors meant with 'pores' and how to recognise these in the pictures. It should be clearly defined how they identify a membrane indent as fusion pore. In case of the hourglass profiles the authors event state that they are 'likely in the process of fusion'. Apart from defining fusion, how do they distinguish between fusion and fission of membranes?

The technique we have used that unambiguously shows pores is image reconstruction from tomograms of whole thick sections. We have made it clearer that the pores are seen in such reconstructions when we describe the EM of hourglass profiles in V7+V8KO cells on Page 8 of the revised manuscript. In the preceding sentence we have tried to make it clearer that whilst we believe that 'hourglass' and 'omega-shaped' profiles of organelles are likely to be in the process of fusion, we cannot rule out fission.

VAMP7/VAMP8 KO phenotype
In VAMP7/VAMP8 KO cells the authors describe hourglass profiles associated with what they identify as 'late endosomes' (Figure 3A). However, these are very empty organelles and in my opinion early endosomes. What are the author's criteria for naming organelles early or late endosomes? VAMP7 and VAMP8 are late endosome -lysosomal SNAREs. Why would KO of VAMP7 and VAMP8 block fusion with early endosomes?

In our revised manuscript, we have expanded the paragraph on Pages 5 and 6, in which we describe the criteria we have used to identify late endocytic organelles in EM sections. We have added a reference to a review by Klumperman and Raposo, 2014 that summarises much of the literature regarding the characteristics of late endosomes and provides a consensus view that the switch from early to late endosomes occurs when as few as 5-8 ILVs accumulate. We have also cited a paper by Sachse et al., 2002 that showed the presence of flat clathrin coats and emanating tubules on late endosomes as well as early endosomes. We have pointed out that early endosomes mature into late endosomes and that an EM section which contains even relatively few ILVs is likely to be a section through a late endosome, although one that has not completed its maturation. We do not know of any evidence from the literature or our own studies suggesting a role for VAMP7 in early endosome fusion, although VAMP8 has been implicated (Antonin W. et al, 2000. Mol. Biol. Cell 11: 3289-3298). Nevertheless, we saw no obvious change in the EEA1 immunofluorescence labelling pattern in any of our three knock out cell lines), consistent with the effects we observed being on late endocytic organelles and not early endosomes.
In figure 3A. it takes quite some goodwill to see the tethers in the EM pictures that are represented in green in the model.
The organelles in Fig. 3C,D (I would say autolysosomes) are definitely a later stage of organelle maturation than the organelles in Figure 3A and B, and they show a different type of tethering (arrays). All in all these data raise too many questions on what the images exactly represent.

We accept that there are many outstanding questions but believe that our description of the tethering is accurate. We agree that it is difficult to see the tethers in the EM tomographic slice shown in previous Fig. 3A (now Fig. 4A in the revised manuscript). This is because the tethers are mainly at the 'back' of the reconstruction of the tomogram of the whole thick section and are more visible in other tomographic slices, which we have shown at higher magnification in what was Fig.3B (now 4B). Movie 2 shows the full reconstruction and from that it is clear that the tethers (green) are not evenly distributed around the edges of pores connecting 'hourglass bulbs' of late endocytic organelles. We agree that the organelles in previous Fig. 3C,D (now 4C,D) are at a later stage of organelle maturation. As we have explained in our manuscript, the observations of the larger arrays of tethers linking these later stage organelles were rare and contrasted with the

© 2021. Published by The Company of Biologists under the terms of the Creative Commons Attribution License (https://creativecommons.org/licenses/by/4.0/).

observations of ‘hourglass’ profiles in which only a few tethers were present, close to the pores seen between organelles.
How do the authors explain the (mildy) altered fluorescent LAMP staining pattern in these cells when there is no obvious effect on endolysosomal morphology?

As we have explained in the first paragraph of our Discussion (revised manuscript Page 12), the EM observations of the V7+V8KO cells showing the accumulation of vesicular LAMP carriers tethered to MVBs, ‘hourglass’ profiles of attached organelles with small pores linking their lumens and tethered organelles at a later stage of maturation taken together can, we believe, account for the increase in size of LAMP1-positive puncta observed by immunofluorescence microscopy i.e. the (mildy) altered fluorescent LAMP1 staining pattern.
Authors claim that there is no alteration of autophagy and no changes in LC3 in VAMP7/VAMP8 KO cells. Both VAMP7 and VAMP8 have been identified several times as players in the autophagy process (fusion of lysosomes-autophagosomes).
Therefore, they should show data to confirm this statement, instead of “data not shown”. The finding is intriguing since it again questions the role of VAMP7/VAMP8 in late endosome – lysosome fusion.

We agree with the reviewer that there are several reports in the literature suggesting that VAMP7 and VAMP8 are players in the autophagy process. Autophagy is not the focus of our paper, so we have removed the references to immunofluorescence microscopy of ubiquitin and LC3. However, we have retained the LC3-II immunoblot now shown in revised supplementary Fig. S1C, which does reveal that there is unlikely to be a major alteration of the autophagy pathway in the V7+V8KO cells. One possibility is that YKT6 is compensating for the loss of VAMP7 and VAMP8 in this pathway but we believe that a full investigation of this issue is beyond the scope of this manuscript, which is focussed on organelle tethering and pore formation
It is interesting that depletion of YKT6 impairs the delivery of Dextran to enzymatically active organelles in VAMP7-VAMP8 KO cells. However, the lack of a proper rescue assay is a gap. Rescue with VAMP7 is not appropriate since this rescues VAMP7 KO and not YKT6 depletion.

We agree with the reviewer that the lack of a YKT6 rescue assay is a gap, but as we have explained in our manuscript it is known that overexpressed mammalian YKT6 does not target correctly to membranes and remains cytosolic thus making rescue experiments challenging. Nevertheless, we do not think it is inappropriate to have rescued the siRNA- mediated knockdown of YKT6 with VAMP7, because as we have explained (revised manuscript Page 12), ‘the yeast orthologue Ykt6p has been shown to be capable of substituting for the R-SNARE Nyv1p (the orthologue of VAMP7) in yeast vacuole fusion (Thorngren et al., 2004) and VAMP7 has been shown capable of substituting for YKT6 in the fusion of autophagosomes with lysosomes in Drosophila (Tak.ts et al.,2018).’
Figure 5A and 5B —why are there only 3 data points? All cells should be represented, not only the averages within an experiment. In general, I don’t think this part is thoroughly done, maybe depletion of YKT6 blocks the endocytosis delivery to lysosomes at an earlier/ different stage in the endocytic pathway?

Although there are different ways of plotting our data, we respectfully submit that plotting the independent means of 3 experiments for each condition (≥30 cells total from 5 fields per condition in each experiment), is acceptable practice. In the revised manuscript the previous Fig. 5A and 5B have not been altered but are renumbered as 7A and 7B.
CHMP6 KO cells
CHMP6 KO cells In the CHMP6 KO cells authors see MVBs tethering to enlarged endo-lysosomes. What is the nature of these MVBs? Since these cells lack the ESCRT complexes involved in the making of intraluminal vesicles.

We have previously shown that neither ESCRT-II nor CHMP6 are required for down- regulation of virally ubiquitinated MHC Class I but instead utilizes a non-canonical pathway requiring HD-PTP, which likely acts as an alternative linker between ESCRT-I and ESCRT- III (Parkinson et al., 2015). In our 2015 paper we showed that MVBs are still formed in the CHMP6-depleted cells and demonstrated by immunoEM that MHC Class I is still delivered to the ILVs. In our 2015 paper we also cited several papers that provide convincing evidence for ESCRT-independent formation of, and sorting into, MVBs.
In CHMP6 KO cells there are many tethering profiles judged by the presence of electrondense

© 2021. Published by The Company of Biologists under the terms of the Creative Commons Attribution License (https://creativecommons.org/licenses/by/4.0/).

staining between compartments. A weakness in the paper is the identification of these electrondense structures. This is done on basis of morphology, the criteria for which are not made clear by the authors and the interpretation of which leaves room for dispute. Authors tried but failed to label Vps18 with pre-embedding EM. I suggest to try immunoEM of cryosections, as they did for Vps35 labeling.

We have certainly tried to use immunoEM on Tokuyasu frozen sections to label tethers in our CHMP6-depleted HeLa cells, but without success to date. Our best VPS18 labelling at EM level has been with the pre-embedding technique. However, as we have explained in our manuscript (Page 10 of revised version), although we could see VPS18 association with membranes, we were unable to see antibody labelling of the tethers themselves, possibly because we were unable to achieve sufficiently high labelling density due to poor antibody access to epitopes embedded in the tightly packed tether arrays (a situation we were unable to improve by over-expressing tagged-VPS18).
Depletion of CHMP6 causes extensive tethering of MVBs to each other and to swollen endolysosomes. Fig. 4B,C and Movie 3 are interesting images, giving a first insight into where pores are formed relative to filament tethering. A next step could by cryo-EM

We are not completely certain what the reviewer is requesting here. We can see the tethers in high pressure frozen thin sections of the CHMP6-depleted cells and in the revised manuscript we have moved these images from the supplementary information into new Fig. 5B. It may be that the reviewer is suggesting that we consider the use of a Titan Krios microscope on appropriately prepared samples to see if we could obtain the types of image that have allowed structural determination of, for example, retromer coats on tubules in the fungus *C. thermophilum*. Such an approach may become possible in the future, but is well beyond the scope of the present study.
In CHMP6-depleted HeLa cells, the human HOPS protein VPS18 associates with swollen LAMP-1 endolysosomes. Depletion of VPS18 by IF prevents formation of these swollen structures. However, the authors do not show if this also prevents the formation of tethers between MVBs and endolysosomes, which would be a key experiment for this line of investigation.

We accept that this would be a good experiment if we could find a good way to do it. Unfortunately, at present we do not have a simple EM assay for the tethers that would easily allow us to quantify directly the amount of tethering in CHMP6-depleted HeLa cells in the presence and absence of VPS18.

Pores are preferentially formed near the edge of tethered regions. Pore expansion is coupled to the release of the filamentous tethers. However, authors cannot show what these tethers are. That would be a strong result.

We agree with the reviewer that it would be a strong result if we could show this. However, as we have explained above, to date we have not been achieved labelling of the tethers by immunoEM.

Reviewer 3 Comments for the Author:
The paper is well written, the data is convincing and the electron microscopy and tomography is spectacular. We thank the reviewer for this assessment of the EM and tomography that we have presented.
The following minor points should however be addressed:
An assay measuring delivery of endocytosed dextran to lysosomes that stain positive for a magic red cathepsin B substrate is used. This assay relies on cathepsin B being delivered to lysosomes, even though this occurs via the endocytic pathway so anything that inhibits delivery of dextran is likely to inhibit delivery of newly synthesised cathepsins too. Given that the MR signal requires the cathepsins to be active then a positive MR signal represents a lysosome that has received cathepsin so clearly there are lysosomes present in VAMP7/8 KO cells that can receive both dextrans and cathepsins. In VAMP7/8 KO cells depleted of YKT6 there is an inhibition of delivery of dextrans to MR positive lysosomes so I don't quite understand how the cathepsin is being delivered if the dextrans are not. Is the turnover of cathepsins sufficiently slow that there is sufficient cathepsin synthesised before knockdown of YKT6 to cleave the MR substrate? I think this point should be discussed/clarified in the text.

We have not studied the delivery of endocytosed cargo other than fluorescent dextran to acid hydrolase+ve endolysosomes in any of our knockout cell lines nor have we examined the delivery of

© 2021. Published by The Company of Biologists under the terms of the Creative Commons Attribution License (https://creativecommons.org/licenses/by/4.0/).

newly synthesised cathepsins. Our assay relies on the presence of cathepsin B in endolysosomes but we cannot rule out a reduction in delivery. To clarify this we have added a sentence to the final paragraph of our Discussion in the revised manuscript (Page
14) explaining that ‘it is clear from the use of the Magic Red® substrate that the endolysosomes in the knockout cells do contain sufficient active Cathepsin B to give a good fluorescence signal even in those experiments in which we also depleted YKT6.’ Although we agree with the reviewer that the depletion of YKT6 might be expected to reduce delivery of newly synthesised cathepsin B, we note that under our depletion conditions this is unlikely to be a complete block because endocytic delivery of fluorescent dextran in our assay is still
~30% or more of that without depletion.
Figure 2B: Clathrin patches are indicated on the tomogram in dark blue but are quite hard to see in the images- maybe it would be more accurate to describe them as ‘resembling the clathrin coats seen on flattened regions of the endosomal limiting membrane’

In our revised manuscript the previous Fig. 2B has become Fig. 3A. Because the clathrin patches on the tomographic slice shown in the left-hand panel of Fig. 3A are hard to see, we have added a further tomographic slice as Fig. 3B, which shows the flat clathrin patches more clearly.
Also, are the authors sure about that the tube like structure on the right of the vacuole in Figure 2B (running parallel to the limiting membrane) should be coloured pink? On the movie it looks like it could be ER to me (especially as it is sitting by a tubular extension of the limiting membrane and ER:endosome contacts are believed to mark sites of recycling tubule scission).

We thank the reviewer for pointing this out. We agree that the tube-like structure on the right of the vacuole in previous Figure 2B (now Fig. 3A in revised manuscript) might be ER and we have now coloured this tubular element in white in the revised figure and in revised Movie 1.

Figure 4A: A lower magnification view would be helpful to see the enlarged endolysosome.

In our revised manuscript the previous Fig.4A has become Fig. 5A. We have replaced the previous left-hand panel with a lower power image of the same enlarged endolysosome.
Summary of Changes to the manuscript Main Text
Results
Figure numbers altered throughout to accommodate panels moved from supplementary figures into main figures and/or additional panels added as indicated in the table below. Page 4. Removal of reference to fewer labelled puncta per cell in the sentence describing the subtle alteration in the distribution of LAMP1 immunofluorescence in VAMP7+VAMP8 double knockout cells.
Page 5. A sentence added to explain why we used VPS33A as a positive control in Fig. 1C. Pages 5&6. First paragraph expanded to explain more clearly criteria used to define late endocytic organelles.

Page 6. A sentence added to explain that vesicle tethers were most clearly observed in tomogram reconstructions/videos and single tomographic slices of EM thick sections.
Page 7. A new paragraph added covering tubules associated with MVBs and incorporating previous description of tubules emanating from MVBs, which had been on the next page. Pages 7&8. Changes to the paragraph on that second morphological change that we observed in the VAMP7+VAMP8 double knockout cells. The changes explain that whilst the ‘hourglass’ and ‘omega-shaped’ profiles observed are likely to be of organelles in the process of fusion, we cannot rule out fission and expand our comments on the location of tethers.
Page 8. The sub-heading ‘Depletion of CHMP6 causes extensive tethering ….’ has been moved up so as to precede the paragraph explaining why we sought a treatment resulting in more extensive tethering and why we re-visited the CHMP6-depletion phenotype. We believe this aids a more logical flow to the narrative.
Discussion
Page14. Addition of sentence explaining that we have not studied the delivery of other cargoes or newly synthesised cathepsins to endolysosomes and commenting on the fact that there is sufficient active Cathepsin B in the endolysosomes to give a good fluorescence signal even after YKT6 depletion.

Materials and Methods.
Pages 15&16. We have added information about non-commercial in-house antibodies that was

© 2021. Published by The Company of Biologists under the terms of the Creative Commons Attribution License (https://creativecommons.org/licenses/by/4.0/).

previously in Supplementary Methods.
Page 16. We have added information about the HeLa cells used that was previously in Supplementary Methods.

References.
Four references added, which are Hasegawa et al. 2003, Hewitt et al. 2002, Klumperman and Raposo, 2014 and Sachse et al. 2002.

Figure legends. Legend supplied for new FIG 3B and left hand (LH) panel of new FIG 5A. Sections of figure legends and supplementary figure legends moved in line with the changes to the figures outlined in the table below:

NEW FIGURES	PREVIOUS FIGURES
FIG 1 A, B, C (no change)	FIG 1A, B, C
FIG 2A FIG 2B FIG 2C FIG 2D FIG 2E FIG 2F	FIG 2A Suppl FIG S2D Suppl FIG S2E Suppl FIG S2F Suppl FIG S2G Suppl FIG S2H
FIG 3A FIG 3B (new image) FIG 3C FIG 3D	FIG 2B Suppl FIG S2A Suppl FIG S3B
FIG 4A, B, C, D (only FIG number changed from 3 to 4 for these four panels)	FIG 3A, B, C, D
FIG 5A (new LH panel) FIG 5B	FIG 4A FIG 4B
FIG 6A	FIG 4C
FIG 6B FIG 6C FIG 6D FIG 6E FIG 6F	Suppl FIG S4A Suppl FIG S4B Suppl FIG S4C Suppl FIG S4D Suppl FIG S4H
FIG 7A, B (only FIG number changed from 5 to 7)	FIG 5A, B

Supplementary Information.

Legend to Movie 1. Addition of a sentence about tubular elements coloured white.

Supplementary Figure legends. Sections of supplementary figure legends moved in line with the changes to the supplementary figures outlined in the table below:

NEW FIGURES	Previous FIGURES
Suppl FIG S1A, B, C, D, E, F, G, H (no change)	Suppl FIG S1A, B, C, D, E, F, G, H
Suppl FIG S2A Suppl FIG S2B	Suppl FIG S2B Suppl FIG S2C
Suppl FIG S3	Suppl FIG S2I
Suppl FIG S4	Suppl FIG S3A

© 2021. Published by The Company of Biologists under the terms of the Creative Commons Attribution License (https://creativecommons.org/licenses/by/4.0/).

Suppl FIG S5A Suppl FIG S5B	Suppl FIG S3C Suppl FIG S3D
Suppl FIG S6A Suppl FIG S6B Suppl FIG S6C	Suppl FIG S4E Suppl FIG S4F Suppl FIG S4G
Suppl FIG S7A, B, C, D, E,<br>F, G (only Suppl FIG number changed from S5 to S7)	Suppl FIG S5A, B, C, D, E, F, G

Supplementary Methods deleted. Information about non-commercial in-house antibodies moved into main Materials and Methods section.

Second decision letter

MS ID#: JOCES/2020/255463

MS TITLE: Organelle tethering, pore formation and SNARE compensation in the late endocytic pathway

AUTHORS: Luther J Davis, Nicholas A Bright, James R Edgar, Michael DJ Parkinson, Lena Wartosch, Judith Mantell, Andrew A Peden, and J. Paul Luzio
ARTICLE TYPE: Research Article

I am happy to tell you that your manuscript has been accepted for publication in Journal of Cell Science, pending standard ethics checks.

Reviewer 1

Advance summary and potential significance to field

This paper makes conceptual advances for the mechanisms underlying kiss and run between organelles, an essential for proper organelle maturation. It shows novel data on pore formation between organelles and underlying mechanisms.

Comments for the author

The authors have taken into account my concerns on the previous version and the revised version is nicely improved.

Reviewer 2

Advance summary and potential significance to field

The revised manuscript has clearly improved in flow and clarity. Not all my comments are addressed, but I accept that the authors did what reasonably can be expected given experimental restraints and considering the scope of the paper.
Although it remains a setback that the identity of the tethers was not established, the high quality of the EM images and the conceptual insights provided by the data do warrant publication in JCS.

© 2021. Published by The Company of Biologists under the terms of the Creative Commons Attribution License (https://creativecommons.org/licenses/by/4.0/).

Comments for the author

Minor comments

Why is it that there is only one LC3 form visible in the blot in Fig S1C, whereas in Fig S5 we see both forms? The ratio between LC3I:II is an informative indicator for autophagic activity

The blot in figure S5 I not only shows an increase in total LC3 but also seems to show a decrease in the LC3I : LC3II ratio (although this should be quantitated to be sure), which is another indicator for increased autophagosome formation but not mentioned by the authors.

<u>Reviewer 3</u>

Advance summary and potential significance to field

In this paper the authors show, surprisingly, that double knockout of two SNAREs, VAMPs 7 and 8, that have previously been implicated in endosome:lysosome fusion, induces a comparatively mild cellular phenotype and does not affect delivery of endocytic cargo to acid hydrolase positive lysosomes. They show that this is because another SNARE, YKT6, compensates for loss of VAMPS 7 and 8. The VAMP7/8 knockout cells do however show morphological abnormalities, specifically an accumulation of vesicles carrying newly synthesised LAMP1 tethered to endosomes and the presence of hourglass profiles where adjacent endosomes and lysosomes are attached by filamentous structures and connected by pores. The authors further investigate the relationship between the pores and tethers by depleting cells of CHMP6, previously shown to accumulate tethered endosomes and lysosomes, and show that pore formation occurs at the edge of tethered regions and suggest that full fusion requires expansion of the pore. The paper is well written, the data is convincing and the electron microscopy and tomography is spectacular. The delivery of cargo to lysosomes for degradation is a fundamental process crucial for cellular homeostasis and yet there is still much that we don't know about this process. I believe this paper would be of wide interest not only to lysosome biologists but also those interested in organelle interaction and membrane fusion in general, since tethering, followed by SNARE-dependent fusion, is a universal mechanism for membrane transport.

Comments for the author

The authors have satisfied all by queries and I recommend that the paper be published.

© 2021. Published by The Company of Biologists under the terms of the Creative Commons Attribution License (https://creativecommons.org/licenses/by/4.0/).
